# Validation of the prognostic gene portfolio, ClinicoMolecular Triad Classification, using an independent prospective breast cancer cohort and external patient populations

**DOI:** 10.1186/bcr3686

**Published:** 2014-07-04

**Authors:** Dong-Yu Wang, Susan J Done, David R Mc Cready, Wey L Leong

**Affiliations:** 1 Department of Surgical Oncology, Princess Margaret Cancer Centre, University Health Network, Toronto, ON M5G 2M9, Canada; 2 Department of Pathology, Princess Margaret Cancer Centre, University Health Network, Toronto, ON M5G 2M9, Canada; 3 Campbell Family Institute of Breast Cancer Research, Princess Margaret Cancer Centre, University Health Network, Toronto, ON M5G 2M9, Canada; 4 Department of Surgical Oncology and Campbell Family Institute of Cancer Research, Princess Margaret Cancer Centre, University Health Network, University of Toronto, 610 University Avenue, Toronto, ON M5G 2M9, Canada

## Abstract

**Introduction:**

Using genome-wide expression profiles of a prospective training cohort of breast cancer patients, ClinicoMolecular Triad Classification (CMTC) was recently developed to classify breast cancers into three clinically relevant groups to aid treatment decisions. CMTC was found to be both prognostic and predictive in a large external breast cancer cohort in that study. This study serves to validate the reproducibility of CMTC and its prognostic value using independent patient cohorts.

**Methods:**

An independent internal cohort (n = 284) and a new external cohort (n = 2,181) were used to validate the association of CMTC between clinicopathological factors, 12 known gene signatures, two molecular subtype classifiers, and 19 oncogenic signalling pathway activities, and to reproduce the abilities of CMTC to predict clinical outcomes of breast cancer. In addition, we also updated the outcome data of the original training cohort (n = 147).

**Results:**

The original training cohort reached a statistically significant difference (p < 0.05) in disease-free survivals between the three CMTC groups after an additional two years of follow-up (median = 55 months). The prognostic value of the triad classification was reproduced in the second independent internal cohort and the new external validation cohort. CMTC achieved even higher prognostic significance when all available patients were analyzed (n = 4,851). Oncogenic pathways Myc, E2F1, Ras and β-catenin were again implicated in the high-risk groups.

**Conclusions:**

Both prospective internal cohorts and the independent external cohorts reproduced the triad classification of CMTC and its prognostic significance. CMTC is an independent prognostic predictor, and it outperformed 12 other known prognostic gene signatures, molecular subtype classifications, and all other standard prognostic clinicopathological factors. Our results support further development of CMTC portfolio into a guide for personalized breast cancer treatments.

## Introduction

Since the first gene expression profile describing the molecular subtypes of breast cancers [1], numerous gene signatures have been developed mainly by answering specific clinical or biological questions, often by dichotomizing the targeted sub-populations into a good and a bad risk group. Many of these gene signatures have claimed prognostic significance in breast cancers, but only a few have been incorporated into clinical practice [2,3]. We recently reported a clinical triad classification system using a genomic approach based on the common gene expression pattern of human epidermal growth factor receptor 2 (HER2) positive and triple negative (HER2+/TN) breast cancers. The 803-gene set termed ClinicoMolecular Triad Classification (CMTC) categorized breast cancers into one of three clinical treatment groups (triad) with prognostic and predictive implications [4]. Based on the gene expression profile from the entire breast cancer genome, CMTC also provides a molecular portfolio of 14 known prognostic gene signatures and 19 oncogenic pathways with the association of many important clinicopathological variables. These portfolios represent unique fingerprints of the biological processes involved which can then be exploited as a guide to target specific pathways for personalized medicine in breast cancer patients. Requiring only a fine needle aspirate from a tumor, CMTC analysis can be performed at the time of initial diagnostic biopsy which offers a unique advantage of early treatment planning, including the use of neo-adjuvant chemotherapy to improve breast conservation in selected patients. As part of the development of CMTC into a clinical tool to guide personalized prognostication and treatment decisions for breast cancer patients, we aimed to validate CMTC using an updated training cohort with a longer follow-up, a second larger and independent prospective cohort and a new external validation cohort to demonstrate that CMTC is reproducible, independent and clinically relevant.

## Methods

### Patients and samples

The study was funded by Genome Canada and the University Health Network, and was approved by institutional research ethics boards at University Health Network and Mount Sinai Hospital (Toronto, ON, Canada). All surgical patients with new breast cancers at the Princess Margaret Cancer Centre of University Health Network and Mount Sinai Hospital were approached and all patients who consented were included. A total of 501 patients with breast tumors were recruited and divided into two internal cohorts in this study. The first 149 consecutive evaluable invasive breast cancers were recruited between 2003 and 2008. They were used as the training cohort in developing the ClinicoMolecular Triad Classification as reported previously [4]. In this study, we updated the clinical status of these patients for an additional two years of follow-up. Two cases were excluded due to loss in follow-up, and the remaining 147 patients were included in the analyses. The second internal cohort included the next 340 consecutive surgical patients with newly diagnosed breast cancers recruited between 2008 and 2010. Fine-needle aspiration biopsies (FNAB) were taken before surgery for all the patients in the second cohort by using the method described previously [4]. FNAB specimens were snap-frozen to -80°C for later processing. After excluding tumors with low RNA yield (n = 8, success rate 97%), non-invasive cancers (n = 27) and insufficient follow-up (n = 21), the remaining 284 invasive breast cancers formed the second internal cohort for the purpose of this validation study. All 501 internal patients and their clinical, pathological and microarray information are tabulated in Table S1 in Additional file 1. The clinical outcome data were updated for all the patients in both internal cohorts until July 2012.

### RNA extraction and microarray experiment

The RNA extraction and microarray experiment of the first internal cohort has been reported previously [4]. The microarray data were generated using version 2 of the genome-wide Illumina Human Ref-8 BeadChip (Illumina Inc, San Diego, CA, USA). For the second internal cohort, the microarray experiment was performed similar to the first cohort except that version 3 of Illumina Human Ref-8 BeadChip (Illumina Inc) was used instead. In brief, the frozen FNAB lysates were thawed and RNAs were extracted with RNeasy Mini Kit (Qiagen, Valencia, CA, USA). The quality and quantity of the RNA were analyzed using an Agilent 2100 Bioanalyzer (Agilent Technologies, Palo Alto, CA, USA). The DNA microarray process was performed according to the Illumina Whole-Genome Gene Expression direct hybridization assay protocols (Illumina Inc) at The Centre of Applied Genomics (Toronto, ON, Canada). First, 250 ng of total RNA were reverse-transcribed into cDNA, followed by *in vitro* transcription amplification to generate biotin-labeled cRNA using the Ambion - Illumina TotalPrep-96 RNA Amplification Kit (Applied Biosystems/Ambion, Austin, TX, USA). Next, 750 ng of the labeled cRNA were hybridized to Illumina Human Ref-8 v3 BeadChip. The scanned Illumina microarray image data were extracted by the Gene Expression Module for Genome Studio V2011.1 (Illumina Inc) using background subtraction and quantile normalization methods for direct hybridization assays.

### Microarray datasets

The microarray data of the two internal breast cancer cohorts (n = 501) is available at the Gene Expression Omnibus website [5] as GEO series GSE16987 for the original internal training set reported previously [4] and GSE45725 for the internal validation set in this current study, respectively. We also used the microarray data of 4,420 breast cancers from two external databases: 1) 2,239 breast cancers reported in the original study by combining 13 external datasets using Affymetix and Agilent platforms [4]; and 2) 2,181 breast cancers from two gene expression datasets using the Illumina platform reported recently from the Gene Expression Omnibus as GEO series GSE22219 [6] and the European Genome-Phenome Archive website [7] as EGA accession number EGAS00000000083 [8]. The data processing for the different microarray platforms has been described previously [4].

### Data analyses and statistics

The classifications of CMTC, scoring and integration of published prognostic gene signatures and oncogenic pathways have been presented previously in detail [4]. The chi-square test and Fisher's exact test were used to test the statistical significance of the clinical and pathological variables between different classifications. Disease-free survival was used to evaluate clinical outcomes including all types of recurrence and breast cancer-specific death. Kaplan-Meier analysis was used to compare patients’ survivals in differential clinical and gene expression groups, and their statistical significances were determined by the Log-rank test. The Cox proportional hazard method was performed for the univariate and multivariate analyses of prognostic factors. Pearson correlation was used to determine the relationships among the gene signatures and oncogenic pathways. All reported *P* values were two-sided, and a *P* value of less than 0.05 was considered statistically significant.

## Results

### Prospective follow-up of the original training cohort

**Figure 1:**
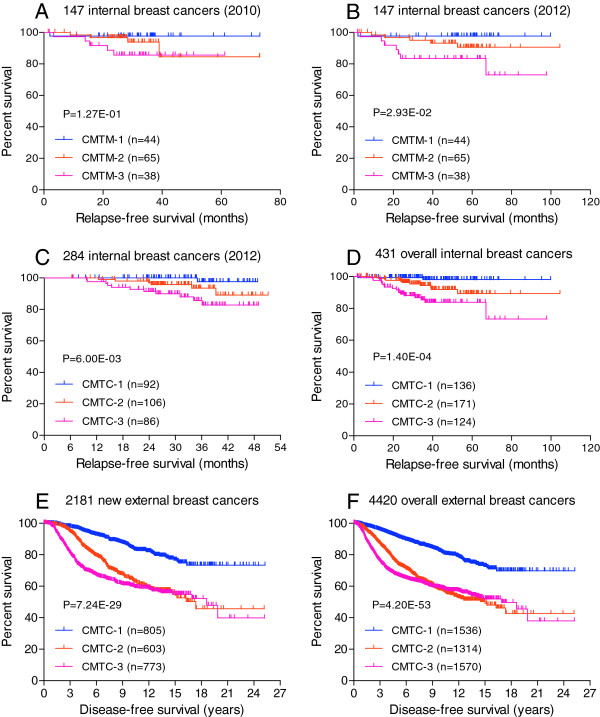
**Kaplan-Meier analyses of relapse-free survivals of the three *CMTC *groups in internal and external breast cancer cohorts. (A)** The 147 patients in the original training cohort with a median follow-up of 30.13 months in 2010. **(B)** The 147 patients in the same training cohort with an updated follow-up of 54.97 months in this study. **(C)** The 284 patients in the new independent internal cohort with a median follow-up of 32.13 months. **(D)** The combined 431 patients in both internal cohorts. **(E)** The 2,181 patients in the new external validation cohort with a median follow-up of 7.45 years. **(F)** The combined 4,420 patients in the two external cohorts from the original study and this study. The *P* values were determined using the log-rank test based on overall comparisons.

We followed all the patients [4] from the original training cohort (n = 149) prospectively between 2010 and 2012 except for two patients who were lost to follow-up due to relocation. The clinical status of the remaining 147 patients was updated prospectively (Figure 1A and B). Compared to our first study (median follow-up = 30 months), a longer median follow-up of 55 months yielded three additional recurrences (one in CMTC-2 and two in CMTC-3) leading to a statistically significant difference (*P* <0.05) in relapse-free survival among the patients in the three CMTC groups (Figure 1B).

### Reproducibility and validation of CMTC in the second internal cohort

**Figure 2:**
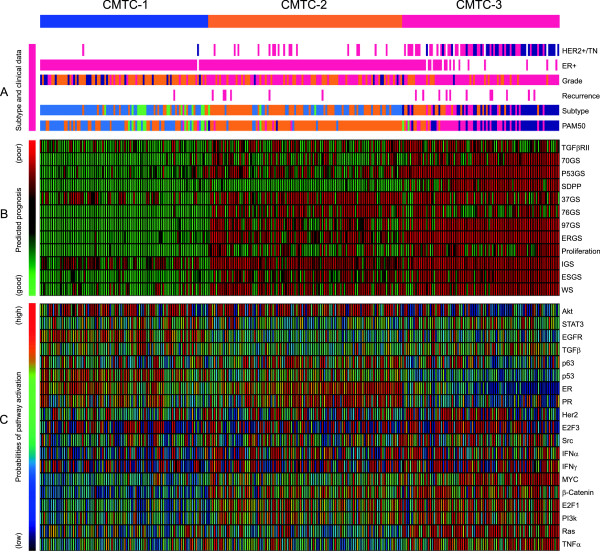
***CMTC *portfolios of 284 breast cancers in the new internal cohort.** The 284 breast cancers are grouped by CMTC with their portfolios shown in columns. Rows presented: **(A)** Tumor receptor status, grade, recurrence and molecular subtypes. The multi-color bars indicate the following: HER2+/TN: HER2+ (human epidermal growth factor receptor 2 positive), deep pink; and TN (triple-negative), dark blue. ER (estrogen receptor): positive, deep pink; negative, empty. Grade: grade 1, dark blue; grade 2, dark orange; and grade 3, deep pink. Recurrence: yes, deep pink; no, empty. The subtype (intrinsic subtype) and PAM50 (prediction analysis of microarray of 50 genes): normal-like, lime; luminal A, blue; luminal B, dark orange; basal-like, dark blue; and Her2+, deep pink. **(B)** The scores of prognostic gene signatures: TGFβRII (type II TGF-β receptor [9]), 70GS (MammaPrint™ [10]), P53GS (mutated P53 GS [11]), SDPP (stroma-derived prognostic predictor [12]), 37GS (lethal phenotype GS [13]), 76GS (Rotterdam signature [14]), 97GS (genomic grade index [15]); ERGS (estrogen-regulated GS [16]), Proliferation (proliferation metagene GS [17]), IGS (invasiveness GS [18]), ESGS (embryonic stem cell–like GS [19]), and WS (Wound-response GS [20]). **(C)** The scores of oncogenic signaling pathways. The abbreviation of pathways: STAT3 (signal transducer and activator of transcription 3), EGFR (epidermal growth factor receptor), TTGFβ (transforming growth factor beta), E2F3 (E2F transcriptional factor 3), IFNα (interferon alpha), IFNγ (interferon gamma), E2F1 (E2F transcriptional factor 1), PI3K (phosphatidylinositol 3-kinase), and TNFα (tumor necrosis factor alpha).

To validate CMTC using a completely different internal cohort, another 284 breast cancers were classified using the expression levels of the CMTC 803-gene set [4] that matched 1,142 probes and 775 genes in the HumanRef-8 v3 array. Similar to the original study, the 284 breast cancers were divided into three similarly sized groups with distinct distributions of clinicopathological parameters including clinical receptor status and tumor grade (Table 1 and Figure 2A). CMTC-1 tumors were mostly estrogen receptor (ER) + (99%) and lower grade (Grade 3 = 14%), CMTC-2 tumors were mostly ER + (100%) and high grade (Grade 3 = 60%), whereas most CMTC-3 tumors were HER2+/TN (80%) and high grade (Grade 3 = 77%). The second internal cohort had a larger number of patients (n = 284) but a similar duration of follow-up (median follow-up = 32 months) as the training cohort during our first study (median follow-up = 30 months). The recurrence rates were statistically different among the groups (Table 1), that is, the lowest in CMTC-1 (1.1%), followed by CMTC2 (5.7%) and CMTC-3 (12.8%). The patients in the CMTC-1 group had a better prognosis than those in the CMTC-2 and CMTC-3 groups (*P* <0.01) as demonstrated by Kaplan-Meier analysis and Log-rank test (Figure 1C). When the two internal cohorts were combined (n = 431, median follow-up = 36 months) the differences in recurrence rates (Figure 1D) became more statistically significant (*P* <0.001).

**Table 1:** CMTC and clinicopathological variables in the internal and external validation cohorts

**Variables**	**Internal validation cohort (n = 284)**				**External validation cohort (n = 2,181)**			
	**CMTC-1 number (%)**	**CMTC-2 number (%)**	**CMTC-3 number (%)**	***P*** **value**	**CMTC-1 number (%)**	**CMTC-2 number (%)**	**CMTC-3 number (%)**	***P*** **value**
Total	92(32.4)	106(37.3)	86(30.3)		805(36.9)	603(27.6)	773(35.4)	
Age								
<50	24(26.1)	34(32.1)	21(24.4)	4.52E-01	156(19.4)	92(15.3)	242(31.3)	4.03E-13
> = 50	68(73.9)	72(67.9)	65(75.6)		649(80.6)	511(84.7)	531(68.7)	
Size								
<=2 cm	56(60.9)	47(44.3)	40(46.5)	4.71E-02	412(51.6)	221(37.1)	294(38.7)	6.01E-09
>2 cm	36(39.1)	59(55.7)	46(53.5)		387(48.4)	374(62.9)	465(61.3)	
LN								
(-)	59(64.1)	68(64.2)	56(65.1)	9.88E-01	499(62.1)	298(49.5)	358(46.6)	6.49E-10
(+)	33(35.9)	38(35.8)	30(34.9)		305(37.9)	304(50.5)	411(53.4)	
Grade								
1	20(21.7)	5(4.7)	2(2.3)	8.34E-17	161(21.4)	30(5.2)	16(2.2)	1.36E-125
2	59(64.1)	37(34.9)	18(20.9)		454(60.3)	253(43.8)	143(19.4)	
3	13(14.1)	64(60.4)	66(76.7)		138(18.3)	294(51.0)	580(78.5)	
ER								
(-)	1(1.1)	0(0.0)	57(66.3)	2.20E-35	34(4.2)	17(2.8)	554(71.7)	4.26E-251
(+)	91(98.9)	106(100.0)	29(33.7)		771(95.8)	586(97.2)	219(28.3)	
HER2+/TN								
No	90(97.8)	89(84.0)	17(19.8)	4.63E-32	776(96.4)	542(89.9)	180(23.3)	2.58E-251
Yes	2(2.2)	17(16.0)	69(80.2)		29(3.6)	61(10.1)	593(76.7)	
Event^a^								
No	91(98.9)	100(94.3)	75(87.2)	5.54E-03	686(85.2)	415(68.8)	494(63.9)	3.15E-22
Yes	1(1.1)	6(5.7)	11(12.8)		119(14.8)	188(31.2)	279(36.1)	

^a^Follow-up event, recurrence in internal validation cohort, and distant relapse and died from the specific disease in the external validation cohort. CMTC, ClinicoMolecular Triad Classification; ER, estrogen receptor; HER2, human epidermal growth factor receptor 2; LN, lymph node status; TN, triple-negative.

### Reproducibility and validation of CMTC in the external validation cohort

The ability of CMTC to predict prognosis of breast cancers was strongly supported by the analyses on the first external validation cohort reported previously [4]. This cohort consisted of 2,239 breast cancers collected from 13 breast cancer datasets using Affymetrix and Agilent microarray platforms, and had a median follow-up of 6.33 years. In this study, we added two recently available Illumina microarray datasets to make up a new validation cohort. The first Illumina external dataset contained gene expression data from 216 breast cancers and had a median follow-up of 10 years [6]. The microarray data were generated by using the Illumina HumanRef-8 v1 BeadChip, in which 916 gene probes (732 genes) matched the CMTC 803-gene set, and they were used for CMTC classification. The second Illumina external dataset contained gene expression data of 1,992 breast cancers [8]. Excluding the cases with missing critical histology and follow-up data, 1,965 breast cancers were included in this study. The patients in this dataset had a median follow-up of 7.15 years, and the microarray data were generated by using Illumina HumanHT-12 v3 Beadchip, in which a total of 1,142 probes (775 genes) matched the CMTC 803-gene set, and they were used for CMTC classification. The two Illumina datasets were combined as our new external validation cohort (n = 2,181) in this study. As shown in Table 1, by using the CMTC classifier described above, the 2,181 breast cancers were divided into three similarly sized groups: CMTC-1 (37%), CMTC-2 (28%) and CMTC-3 (35%). The clinical and pathological profiles of the three CMTC groups were very similar to our internal cohorts and the original external cohort: CMTC-1 tumors were mostly ER + (96%) and low grade (Grade 3 = 18%), CMTC-2 tumors were mostly ER + (97%) and high grade (Grade 3 = 51%), whereas CMTC-3 tumors were mostly HER2+/TN (77%) and high grade (Grade 3 = 79%). Using Kaplan-Meier analysis for relapse-free survivals (Figure 1E), the patients in CMTC-1 had a much better prognosis than those in CMTC-2 and CMTC-3 (*P* = 7.24E-29). The difference in relapse-free survivals (Figure 1F) was even more pronounced (*P* = 4.20E-53) when we combined all the patients from the original external validation cohort and the new external validation cohort (n = 4,420).

### CMTC and gene expression prognostic signatures

When we created the CMTC signature to predict the outcome of breast cancers by using gene expression profiling of the training set, we excluded 501 genes that overlapped with 14 published prognostic gene signatures and two gene sets for molecular subtypes in the original study to eliminate any potential confounding effects with these prognostic signatures. Finally, the gene expression profile of the resulting CMTC gene set still matched and outperformed these known independent gene signatures [4]. In this study, the CMTC prognostic framework with 12 microarray-based gene signatures [9-20] was reproduced in the internal validation cohort with 284 breast cancers (Figure 2B). Similar to the training cohort, most CMTC-3 tumors in the validation cohort were classified as ‘poor prognosis’ based on the scores of the 12 prognostic gene signatures, and most CMTC-1 tumors were categorized as ‘good prognosis’. In both the internal training cohort [4] and the validation cohort (Figure 2B), the CMTC-3 group was closely correlated to gene signatures 70GS, P53GS and SDPP using pairwise analyses (Figure S1A and S1B in Additional file 2). Based on Cox proportional analyses of CMTC, receptor status and all other prognostic factors, we observed the highest hazard ratio (HR) when we compared CMTC-1 versus CMTC-3 (HR = 12.55, *P* <0.05), followed by IGS good versus IGS poor (HR = 11.30, *P* <0.05) and CMTC-1 versus CMTC-2 plus CMTC-3 (HR = 8.79, *P* <0.05) in the 284 breast cancers from the new internal cohort. In the 2,181 breast cancers from the new external cohort, the highest HR was found when we compared CMTC-1 versus CMTC-3 (HR = 3.28, *P* <0.01), followed by ERGS good versus ERGS poor (HR = 3.06, *P* <0.01) and CMTC-1 versus CMTC-2 plus CMTC-3 (HR = 2.90, *P* <0.01) (Table S2 in Additional file 2). CMTC also had the highest HR in the original external validation cohort among the 12 known gene signatures as prognostic indicators for breast cancer relapse [4]. These results demonstrated the reproducibility of CMTC as one of the best independent prognostic predictors when compared to these 12 other known prognostic gene signatures.

### CMTC and oncogenic pathway activities

Each CMTC group displayed a distinct pattern of oncogenic pathway activities [21,22] as reported in the internal training cohort before [4]. In this study, this oncogenic pathway pattern in CMTC classes was clearly reproduced in the second internal cohort (Figure 2C): the highest activities in oncogenic pathways Myc, E2F1, Ras and β-catenin were seen again in the CMTC-3 group, with the worst outcome, as shown in the original study. As well, high activities of HER2, TNF and IFN pathways were also observed in the CMTC-3 group. Conversely, ER, progesterone receptor (PR) and wild-type p53 pathways had the lowest activity in CMTC-3 breast cancers. This portfolio of pathway activities was completely opposite to that of CMTC-1, in which the breast cancer patients had a better prognosis. CMTC-2 was distinct from the other two groups with high or moderate activities in most of the pathways that differentiated CMTC-1 from CMTC-3. In a pairwise analysis, ER, PR and wild-type p53 pathways had the highest correlation with CMTC-1; in contrast, Myc, E2F1, Ras and β-catenin pathways were closely related to CMTC-3 in both internal cohorts (Figure S1C and S1D in Additional file 2). These results confirmed that the three CMTC groups have distinct molecular signatures and activities in the selected oncogenic pathways.

### CMTC, receptor status and molecular subtypes

**Figure 3:**
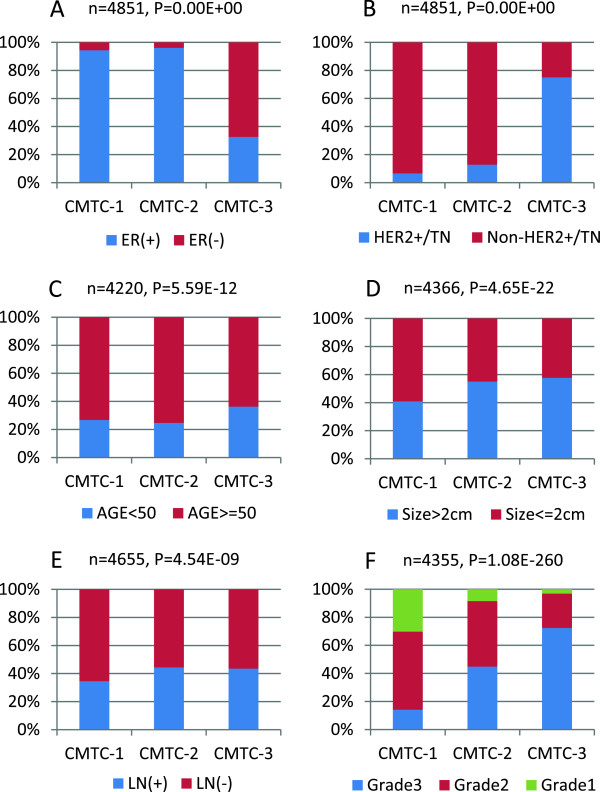
**The association between clinicopathological variables and the three *CMTC *groups in the 4,851 overall breast cancers. (A)** ER status, **(B)** HER2/TN status, **(C)** patient age, **(D)** tumor size, **(E)** lymph node status and **(F)** tumor grade. The *P* values were determined using the Chi-square test and Fisher's exact test. CMTC, ClinicoMolecular Triad Classification; ER, estrogen receptor; HER2, human epidermal growth factor receptor 2; TN, triple negative.

CMTC was originally derived from the molecular phenotype of HER2+ and TN breast cancers [4]. In this study a strong correlation between the three CMTC groups with tumor receptor status is again reproduced in the second internal and external cohorts (Table 1), and in 4,851 available breast cancers by combining all the internal and external cohorts (Figure 3A and B). While both gene signatures for classical intrinsic subtype [23] and PAM50-based subtypes [24] correlated well with CMTC groups, PAM50-based subtypes had a stronger correlation with CMTC than the classical intrinsic subtype: most normal-like and luminal A subtypes were found in CMTC-1; most of luminal B tumors were found in CMTC-2; and most HER2 and basal-like (similar to TN) were found in CMTC-3 in both the internal training cohort [4] and the internal validation cohort (Figure 2A). PAM50 classification also had a higher HR than the classical intrinsic subtype to predict recurrence in the 284 internal and the 2,181 external breast cancer patients (Table S2 in Additional file 2). We wanted to know if the prognostic significance of CMTC can be replicated using PAM50 molecular subtypes by simply grouping normal-like and luminal A subtypes into group 1, luminal B into group 2 and HER2+ and basal-like into group 3. Kaplan-Meier analyses of all available patients (n = 4,851) showed that CMTC remained the most powerful prognostic predictor (Figure S2 in Additional file 2) for disease-free survival (*P* = 2.04E-55) compared to the three PAM50 based groupings (*P* = 5.11E-54), HER2/TN status (*P* = 9.30E-26) and ER status (*P* = 2.40E-20). Based on Cox proportional analysis, we obtained the highest HR when we compared CMTC-1 to CMTC-2 (HR = 1.91, *P* = 2.20E-12), and CMTC1 to CMTC-3 (HR = 1.86, *P* = 1.60E-08) followed by PAM-50, HER2/TN and ER status. CMTC was also found to be an independent predictor of relapse-free survival when we controlled for receptor status (ER- versus ER+ cancers, and non-HER2+/TN versus HER2+/TN cancers) as shown in Figure S3 in Additional file 2.

### CMTC and clinicopathological variables

As in our previous study [4], this study showed that CMTC-1 was again correlated with low tumor grade, and CMTC-2 and CMTC-3 were associated with high grade in both the second internal cohort (Figure 2A) and the new external validation cohort (Table 1). Patient’s age, tumor size and lymph node status were not associated with CMTC in the internal validation cohort of the 284 breast cancers, but these variables became statistically significant in the new larger external validation cohort of 2,181 breast cancers: more younger patients were found in CMTC-3 than in CMTC1-1 and CMTC-2; more patients with a larger tumor size or lymph node positive disease were found in CMTC-2 and CMTC-3 than in CMTC-1 (Table 1). The association of these clinical variables with CMTC was more pronounced when all 4,851 available breast cancers were examined (Figure 3C-3F). To understand the association of these clinicopathological variables with the prognostic significance of CMTC classification, all 3,963 breast cancers with complete relevant clinicopathological and follow-up data in the overall cohort were analyzed (Figure S4 in Additional file 2). Kaplan-Meier analysis and Log-rank test verified that CMTC (*P* = 6.61E-45) had the highest predictive power for relapse-free survival when compared to tumor size (*P* = 3.31E-31, ranked in 2nd), nodal disease and patient’s age. The CMTC classifier remained an independent predictor of relapse-free survival when we controlled for tumor size, nodal status and patient’s age (Figure S5 in Additional file 2). Table 2 shows the results of the multivariate Cox proportional hazards analyses comparing CMTC to receptor status and other clinicopathological variables. Tumor grade and tumor size had higher HR ratios than receptor status; however, the patients in CMTC-2 (HR = 2.06, *P* = 1.00E-15) and CMCT-3 (HR = 2.01, *P* = 3.30E-10) have the highest HRs compared to the patients in CMTC-1.

**Table 2:** Multivariate Cox proportional hazards analyses of clinicopathological variables and CMTC as prognostic indicators in the 3,963 overall breast cancers

**Variables**	**Hazard ratio**	**95%CI**	***P*** **value**
ER (+) versus (-)	1.10	0.89 to 1.36	4.00E-01
Non-HER2/TN versus HER2+/TN	1.26	1.03 to 1.54	2.20E-02
Age <50 versus > = 50	0.85	0.74 to 0.96	1.10E-02
Size < =2 cm versus >2 cm	1.78	1.56 to 2.02	0.00E + 00
LN (-) versus (+)	0.68	0.60 to 0.77	8.20E-10
Grade1 versus 2	1.50	1.17 to 1.91	1.30E-03
Grade1 versus 3	1.40	1.09 to 1.81	9.80E-03
CMTC-1 versus 2	2.06	1.73 to 2.46	1.00E-15
CMTC-1 versus 3	2.01	1.62 to 2.50	3.30E-10

CI, confidence interval; CMTC, ClinicoMolecular Triad Classification; ER, estrogen receptor; HER2, human epidermal growth factor receptor 2; LN, lymph node status; TN, triple-negative.

### CMTC and microarray platforms

The microarray data from external datasets were generated by using one of the three major commercial microarray platforms: Affymetrix GeneChip, Agilent oligonucleotide microarray and Illumina BeadChip. In the previous study, the Illumina platform was used in our training cohort, whereas Affymetrix and Agilent platforms were used in the external validation cohort [4]. In this study, the Illumina platform was used in our second internal cohort and the two newly published external breast cancer datasets. To examine the effects of the microarray platform on CMTC and its prognostic significance, we divided all 4,851 breast cancers based on their microarray platforms and assessed the clinical outcomes by their CMTC classification. We observed the same pattern of clinical outcome among the CMTC groups (better in CMTC-1 and worse in CMTC-2 and CMTC-3) across all three platforms. The prognostic effects and profiles were also comparable among the three microarray platforms (Figures S6A-S6C in Additional file 2). To demonstrate that CMTC can be applied across different commercial microarray platforms, we observed comparable prognostic profiles in 2,449 randomly selected breast cancers (Figure S6D in Additional file 2) and in all 4,851 breast cancers (Figure S2D in Additional file 2).

## Discussion

CMTC was developed to provide a comprehensive test to guide personalized breast cancer treatments. DNA microarray was used as it was the first molecular technology capable of surveying the entire genome reproducibly. While newer technologies exist, such as next-generation sequencing (NGS), it is undeniable that DNA microarray technology is much more mature, low cost and simpler in terms of data storage and analysis, when compared to NGS. Furthermore, there is much more DNA microarray data in the public databases available for independent validation. With a genomic approach, CMTC can provide much more information than other commercially available gene signatures, such as Mammaprint™ [10], that only examine subsets of genes. Our recent study showed that CMTC correlated with many clinical and biological variables known to have prognostic significance in breast cancers. Although the prognostic significance of CMTC was only demonstrated in the first external validation cohort (n = 2,239), the relapse-free survivals in the 149 training cases were not statistically significant due to a low event rate with a median follow up of 31 months [4]. In this study, we prospectively followed the training cohort for an additional two years. Figure 1A and B showed that by having a longer follow-up in the training cohort (median follow up of 55 months), CMTC reached a statistically significant difference in relapse-free survival among the CMTC groups. As in the external cohort in the first study [4], the patients in CMTC-1 had a better relapse-free survival than the patients in CMTC-2 or CMTC-3. To further validate the prognostic significance of CMTC, we used another set of breast cancer patients as an independent internal cohort and a new external cohort in this study. We observed a comparable prognostic significance in the new internal cohort with 284 breast cancers (Figure 1C) and the new external cohort with 2,181 breast cancers (Figure 1E). The prognostic significance of CMTC can also be reproduced in 431 overall internal breast cancers (Figure 1D), and in 4,420 overall external breast cancers (Figure 1F), as well in 4,851 of all available breast cancers by combining all internal and external cohorts (Figure S2D in Additional file 2). Thus, the prognostic significance of CMTC can be reproduced in different independent microarray gene expression datasets.

The gene expression pattern of the CMTC profile can also be used to correlate with independently developed prognostic gene signatures and oncogenic pathway activities as in our previous study [4]. All the gene signatures predicted a poor prognosis in either CMTC-3 alone, or in both CMCT-2 and CMTC-3, but rarely in CMTC-1 (Figure 2B). In both the internal training cohort and the validation cohort (Figure S1A and S1B in Additional file 2), pairwise correlation analyses showed CMTC-3 centroid values were most closely correlated to the scores of prognostic gene signatures 70GS [10], P53GS [11] and SDPP [12]. Cox proportional analysis of the new internal (n = 284) and external (n = 2,181) breast cancer cohorts (n = 431) (Table S2 in Additional file 2), yielded the highest HR between CMTC-1 and CMTC-3 when we analyzed CMTC and all other prognostic gene signatures. This was the first time that we have demonstrated that CMTC was the best predictor of relapse-free survival among all other prognostic gene signatures using prospective data. To ensure that the prognostic significance of CMTC was not confounded by the expression levels of genes that were part of other prognostic gene signatures, we removed all 501 overlapping genes with 14 known prognostic gene signatures (including two non-microarray-based gene signatures) and two gene signatures for molecular subtypes [4] and hence, none of the 803 genes in the CMTC signature can be found in these gene signatures. In this study, once again, we have confirmed that CMTC was reproducible and it was an independent prognostic factor from those known gene signatures using a number of independent breast cancer datasets. We also showed that the CMTC-3 group had the highest activities in the oncogenic pathways Myc, E2F1, Ras and β-catenin with higher activities in HER2, TNF and IFN pathways (Figure 2C). The scores of these pathway activities are most closely correlated with CMTC-3 centroid values in both internal cohorts (Figure S1C and 1D in Additional file 2). Conversely, the scores of oncogenic pathway activity of ER, PR and wild-type p53 were the lowest in CMTC-3 group. These results suggest that the gene expression pattern of CMTC-3 with the worst clinical outcome can be linked to specific networks of oncogenic pathways which may help us to understand the molecular derangements in these cancers.

Using an unsupervised hierarchical clustering analysis of genome-wide expression microarray data, the classical molecular classification divided breast cancers into five intrinsic subtypes: normal-like, luminal A, luminal B, HER2+ and basal-like subtypes [23]. The PAM50 classification was later developed using a training set of breast cancers with known molecular intrinsic subtypes (supervised) to select 10 genes from each of the 5 intrinsic subtypes so that quantitative reverse transcriptase polymerase chain reactions of the 50 genes could be used to reproduce the molecular classification [24]. Recently, PAM50 has been commercialized using the NanoString platform under the trade name Progsigna (NanoString Technologies Inc, Seattle, WA, USA). For comparison purposes, PAM50 classifications in this study were done using the available genome-wide microarray data rather than the NanoString platform. Although likely representative, we understand that this is one of the limitations of our study as comparison across different molecular technology platforms (NanoString and Illumina) can be problematic. Interestingly, we observed that the PAM50 subtype appeared to be more comparable to CMTC both in the internal training cohort [4] and in the internal validation cohort than the classical molecular classification (Figure 1A): most normal-like and luminal A subtypes were found in the CMTC-1 group; most luminal B were found in the CMTC-2 group; and HER2 and basal-like were found in the CMTC-3 group. Because the normal-like subtype has been regarded as normal ‘contamination’ in the tumor specimen, it has been removed from PAM50-based classification [24,25]. In CMTC, HER2+ and TN (similar to basal-like subtype) were grouped together in CMTC-3, and most normal-like and luminal A subtypes were grouped together in CMTC-1. Here, we report that the CMTC can predict clinical outcome better than the classical molecular subtype classification in the 284 internal and 2,181 external breast cancers (Table S2 in Additional file 2) and in the 4,851 overall breast cancers (Figure S2C and S2D in Additional file 2).

CMTC was shown to have a close association with clinical receptor status and a number of clinicopathological variables in the previous study [4]. In this study, the association has been reproduced in the internal validation cohort and the new external validation cohort (Table 1), as well in the 4,851 overall breast cancers (Figure 3). CMTC-1 breast cancers were generally ER+, smaller tumor size, low grade and node negative; CMTC-2 breast cancers were ER+, but larger tumor size, higher grade and more nodal disease; and CMTC-3 breast cancers are more commonly found in younger patients, HER2+/TN, larger tumor size, and more nodal disease.

Comparing with the receptor status of ER, PR, HER2, TN and HER2+/TN, CMTC was proven to be the best prognostic indicator in the 284 internal and 2,181 external patients (Table S2 in Additional file 2). The superiority of CMTC prognostic prediction can be further demonstrated in the analysis using the 4,851 overall patients in the multivariate models (Figure S2 in Additional file 2). The prognostic significance of CMTC remained very strong even if we divided the breast cancers into ER- or ER+ tumors, and non-HER2+/TN or HER2+/TN tumors (Figure S3 in Additional file 2).

Using 3,936 patients with complete clinicopathological data, factors such as younger patient age, larger tumor size, positive node disease and higher tumor grade were associated with an increased risk of poor clinical outcome in Kaplan-Meier analyses (Figure S4 in Additional file 2) and in multivariate analyses using Cox proportional hazards (Table 2). However, CMTC-2 and CMCT-3 groups were found to have the worst outcome when compared to all these clinical and pathological factors. CMTC remained the strongest prognostic predictor even when we controlled for age, and tumor size, tumor grade and nodal status (Figure S5 in Additional file 2). These results clearly show that the prognostic significance of CMTC is independent of these clinical and pathological factors.

A number of gene signatures for breast cancer are commercially available to predict prognosis in specific patient populations using central laboratory facilities [2,3]. In this study, we were able to reproduce the CMTC using three different major commercial microarray platforms and independent external datasets (Figure S6 in Additional file 2). We chose to use a genome-wide microarray platform because we believe that this approach can provide breast cancer patients more comprehensive information on prognosis, treatment prediction and pathway patterns for personalized medicine than other commercial gene signatures. Once the genome-wide gene expression data is collected, it can be used for personalized medicine and it can also be anonymized and deposited into public databases to help us understand the disease better.

In the previous study, we proposed that CMTC could also be used as a platform to personalize treatments: CMTC-1 breast cancers in general can be treated with surgery and endocrine therapy alone; CMTC-2 breast cancers may require additional treatments such as chemotherapy; and neo-adjuvant chemotherapy should be considered for CMTC-3 tumors [4]. Our future study will aim to verify the prediction of treatment outcomes using CMTC classification.

## Conclusions

Our study demonstrates the reproducibility of CMTC and its prognostic significance in both internal and external validation cohorts. CMTC has been shown to be an independent prognostic predictor. It outperformed 12 other known prognostic gene signatures and subtypes, and all standard clinicopathological factors. The robustness of CMTC, independent prediction of clinical outcome, and the ability to provide a portfolio of equivalent scores of different gene signatures and oncogenic pathway activities can potentially make CMTC a useful tool to find novel treatment strategies and provide a biological basis to guide personalized breast cancer treatments. The ability to use fine needle aspiration biopsy to generate a CMTC portfolio prior to surgery gives it a distinct advantage over other currently available prognostic gene signatures.

## Abbreviations

CMTC: ClinicoMolecular Triad Classification; ER: estrogen receptor; FNAB: fine-needle aspiration biopsy; Her2: human epidermal growth factor receptor 2 (also known as ERBB2); HR: hazard ratio; IFN: interferon; PR: progesterone receptor; TN: triple-negative (ER-/PR-/Her2-); TGF: transforming growth factor; TNF: tumor necrosis factor.

## Competing interests

The authors declare that they have no competing interests.

## Authors’ contributions

DYW and WLL designed the project and analyzed the data. DRM and WLL contributed to the collection of clinical material. SJD was the associated pathologist. The manuscript was prepared by DYW and WLL, and proofread by SJD and DRM. All authors read and approved the final manuscript.

## Supplementary Material

Additional file 1: Table S1 Patient information and tumor pathological data of 501 breast tumors in internal training cohort (GSE16987) and internal validation cohort (GSE45725). Click here for file

Additional file 2: Table S2 Comparison of Cox proportional hazards in receptor status, CMTC, subtype and other gene expression signatures as prognostic indicators for recurrence in the 284 internal and 2,181 external breast cancer patients. **Figure S1.** Pairwise comparisons of *CMTC* with gene signatures and oncogenic signaling pathway activities between the internal training cohort and validation cohort. **Figure S2.** Comparison of the prognostic significance between receptor status, molecular subtypes and *CMTC* in the 4,851 overall breast cancers. **Figure S3.** The assessment of the prognostic significance of CMTC among different groups based on the receptor status in the 4,851 overall breast cancers. **Figure S4.** The assessment of the prognostic significance of CMTC among different groups based on other clinicopathologic variables in the 3,963 breast cancers with complete clinical information. **Figure S5.** The assessment of the prognostic significance of CMTC among different groups based on different clinical variable status in the 4,851 overall breast cancers. **Figure S6.** The assessment of the prognostic significance of CMTC among different groups based on the different microarray platforms in the 4,851 overall breast cancers. Click here for file
