# Supplementary material for: Validation of the prognostic gene portfolio, ClinicoMolecular Triad Classification, using an independent prospective breast cancer cohort and external patient populations
Source: Breast Cancer Res. 2014 Jul 4;16(4):R71. doi: 10.1186/bcr3686 (PMC4226941; doi:10.1186/bcr3686)
Supplement: Additional file 1: Table S1 — Patient information and tumor pathological data of 501 breast tumors in internal training cohort (GSE16987) and internal validation cohort (GSE45725). [file bcr3686-S1.pdf]

Supplementary Table S1. Patient information and tumor pathological data of 501 breast tumors in internal training cohort (GSE16987) and internal validation cohort (GSE45725)

Sample_title	GEO_series_accession	GEO_platform_accession	GEO_sample_accession	GEO_raw_data/rep	Sample_type/rep	Sample_RIN/rep	Patient_age	Surgical_margin (cm)	Tumor_type	Tumor_size(cm)	Tumor_grade	Positive_LN(total_LN)	LVI	EIC	ER	PR	HER2	CMTC	Recurrence	Recurrence-free_months	Used_in_the_431BC_dataset?
Breast tumor 1	GSE16987	GPL6104	GSM425140	1825371052_B/1699538091_H	FNA/FNA	6.2/6.2	42	0.018	IDC	1.5	2	0(15)	(-)	(-)	(+)	(-)	(-)	3	0	74.07	yes
Breast tumor 2	GSE16987	GPL6104	GSM425141	1825371052_C	FNA	8.7	56	0.45	IDC/Lobular	2.2	3	0(3)	(-)	(-)	(-)	(-)	(-)	2	0	39.47	yes
Breast tumor 3	GSE16987	GPL6104	GSM425142	1825371052_D	FNA	7.7	40	0.3	IDC	1.5	2	0(7)	(-)	(-)	(+)	(+)	(-)	1	0	32.77	yes
Breast tumor 4	GSE16987	GPL6104	GSM425143	1825371052_E	FNA	7.0	46	0.19	IDC	2.6	2	0(5)	(-)	(-)	(+)	(+)	(-)	1	0	70.93	yes
Breast tumor 5	GSE16987	GPL6104	GSM425144	1825371085_B	FNA	7.1	44	1	DCIS/IDC	2.1	2	0(6)	(+)	(+)	(+)	(+)	(-)	NA	0	31.93	no
Breast tumor 6	GSE16987	GPL6104	GSM425145	1825371085_C	FNA	7.3	63	1.1	IDC	1.8	1	0(3)	(-)	(-)	(+)	(-)	(-)	1	0	67.50	yes
Breast tumor 7	GSE16987	GPL6104	GSM425146	1825371085_D	FNA	8.4	47	0.32	IDC	4	3	8(18)	(+)	(+)	(-)	(-)	(+)	3	0	68.47	yes
Breast tumor 8	GSE16987	GPL6104	GSM425147	1825371085_E	FNA	8.7	48	0.46	IDC	1.9	2	2(11)	(-)	(-)	(+)	(+)	(-)	2	0	71.63	yes
Breast tumor 9	GSE16987	GPL6104	GSM425148	1825371086_B	FNA	7.1	51	1.2	IDC	2.7	3	2(20)	(+)	(-)	(+)	(-)	(-)	2	1	52.63	yes
Breast tumor 10	GSE16987	GPL6104	GSM425149	1825371086_C	FNA	7.2	72	0.2	IDC	3	3	0(1)	(-)	(-)	(+)	(+)	(-)	2	1	8.93	yes
Breast tumor 11	GSE16987	GPL6104	GSM425150	1825371086_D	FNA	7.2	84	0.4	IDC	2.1	1	0(1)	(+)	(-)	(+)	(+)	(-)	1	0	49.57	yes
Breast tumor 12	GSE16987	GPL6104	GSM425151	1825371086_E	FNA	7.4	72	0.4	IDC	1.5	1	0(2)	(-)	(-)	(+)	(+)	(-)	1	0	55.90	yes
Breast tumor 13	GSE16987	GPL6104	GSM425152	1825371087_B	CBX	8.2	58	0.075	IDC	3.5	2	1(17)	(-)	(-)	(+)	(-)	(-)	3	0	71.63	yes
Breast tumor 14	GSE16987	GPL6104	GSM425153	1825371087_C	CBX	7.6	49	0.4	IDC	3.6	2	0(4)	(-)	(+)	(+)	(+)	(-)	1	0	73.13	yes
Breast tumor 15	GSE16987	GPL6104	GSM425154	1825371087_D	FNA	8.3	43	0.3	IDC	2.9	3	1(4)	(+)	(-)	(-)	(-)	(-)	3	0	64.17	yes
Breast tumor 16	GSE16987	GPL6104	GSM425155	1825371087_E	CBX	8.1	73	1	IDC	2.8	3	2(20)	(-)	(-)	(+)	(-)	(-)	2	0	61.03	yes
Breast tumor 17	GSE16987	GPL6104	GSM425156	1825371097_B	FNA	7.5	31	1	IDC	3.5	3	7(16)	(+)	(-)	(+)	(-)	(+)	3	0	71.53	yes
Breast tumor 18	GSE16987	GPL6104	GSM425157	1825371097_C	FNA	8.7	67	0.1	IDC	2	2	1(19)	(+)	(-)	(+)	(+)	(-)	2	0	67.27	yes
Breast tumor 19	GSE16987	GPL6104	GSM425158	1825371097_D	FNA	9.1	45	0.1	IDC	2.8	3	0(3)	(-)	(-)	(-)	(-)	(-)	3	0	22.77	yes
Breast tumor 20	GSE16987	GPL6104	GSM425159	1825371097_E	FNA	9.0	46	0.1	IDC	2.8	3	0(3)	(-)	(-)	(-)	(-)	(-)	3	NA	NA	no
Breast tumor 21	GSE16987	GPL6104	GSM425160	1825371099_B	CBX	9.1	46	0.6	IDC	0.8	1	0(3)	(-)	(+)	(+)	(+)	(-)	1	0	62.53	yes
Breast tumor 22	GSE16987	GPL6104	GSM425161	1825371099_C	CBX	9.0	68	0.65	IDC/Papilloma	1.4	2	0(2)	(-)	(-)	(+)	(+)	(-)	1	0	62.30	yes
Breast tumor 23	GSE16987	GPL6104	GSM425162	1825371099_D	FNA	8.7	51	0.2	IDC	1.4	1	0(2)	(-)	(-)	(+)	(+)	(-)	1	0	69.13	yes
Breast tumor 24	GSE16987	GPL6104	GSM425163	1825371099_E/1699538082_A	FNA/FNA	8.1/7.7	80	0.3	IDC	2	3	0(1)	(-)	(-)	(-)	(-)	(-)	3	1	3.27	yes
Breast tumor 25	GSE16987	GPL6104	GSM425164	1699538082_C	FNA	9.4	46	0.4	IDC	2	2	0(2)	(-)	(+)	(+)	(+)	(+)	1	0	21.50	yes
Breast tumor 26	GSE16987	GPL6104	GSM425165	1699538082_E/1699538082_F	FNA/FNA	8.3/8.0	48	0.12	IDC/lobular	2.1	2	0(1)	(-)	(-)	(+)	(+)	(-)	1	0	68.87	yes
Breast tumor 27	GSE16987	GPL6104	GSM425166	1699538082_G	FNA	7.8	69	1.2	IDC	3.3	1	4(23)	(-)	(-)	(+)	(+)	(-)	1	0	61.33	yes
Breast tumor 28	GSE16987	GPL6104	GSM425167	1699538083_A	FNA	7.0	62	NA	Benign	NA	NA	0(3)	NA	NA	NA	NA	NA	NA	0	0.47	no
Breast tumor 29	GSE16987	GPL6104	GSM425168	1699538083_B	FNA	6.8	45	0	IDC/lobular	4.2	3	1(25)	(+)	(-)	(+)	(+)	(-)	1	0	59.07	yes
Breast tumor 30	GSE16987	GPL6104	GSM425169	1699538083_C/1699538083_D	FNA/FNA	7.3/8.9	52	0.29	IDC	2.8	2	0(1)	(+)	(-)	(+)	(-)	(-)	2	0	68.67	yes
Breast tumor 31	GSE16987	GPL6104	GSM425170	1699538083_E/1699538083_F	FNA/FNA	8.6/8.5	29	0.6	IDC	1.9	3	0(4)	(-)	(-)	(-)	(-)	(-)	3	0	66.73	yes
Breast tumor 32	GSE16987	GPL6104	GSM425171	1699538083_G	FNA	6.2	44	0.2	IDC	2.3	2	1(16)	(+)	(-)	(+)	(-)	(-)	2	0	59.33	yes
Breast tumor 33	GSE16987	GPL6104	GSM425172	1699538089_A	FNA	8.4	56	0.65	IDC	2.5	3	13(28)	(+)	(-)	(+)	(+)	(-)	2	0	6.87	yes
Breast tumor 34	GSE16987	GPL6104	GSM425173	1699538089_B	FNA	7.2	57	0.4	IDC	1	2	8(35)	(-)	(-)	(+)	(-)	(-)	2	0	36.20	yes
Breast tumor 35	GSE16987	GPL6104	GSM425174	1699538089_C	Tissue	6.5	50	0.5	IDC	3.5	2	2(17)	(+)	(-)	(+)	(+)	(-)	1	NA	NA	no
Breast tumor 36	GSE16987	GPL6104	GSM425175	1699538089_D	Tissue	7.3	70	0.3	IDC	3	2	42(44)	(+)	(-)	(+)	(-)	(-)	2	0	104.57	yes
Breast tumor 37	GSE16987	GPL6104	GSM425176	1699538089_E	Tissue	5.8	61	0	IDC	2.4	2	2(18)	(-)	(-)	(+)	(-)	(+)	2	1	39.00	yes
Breast tumor 38	GSE16987	GPL6104	GSM425177	1699538089_F	Tissue	7.8	63	0.8	IDC	2.3	3	0(18)	(-)	(-)	(+)	(-)	(-)	1	0	61.10	yes
Breast tumor 39	GSE16987	GPL6104	GSM425178	1699538089_G	Tissue	7.6	59	2.5	IDC	4	3	1(22)	(-)	(-)	(+)	(-)	(-)	2	1	15.67	yes
Breast tumor 40	GSE16987	GPL6104	GSM425179	1699538090_A	Tissue	6.0	65	0.03	IDC	2.7	3	4(17)	(+)	(-)	(+)	(-)	(+)	2	0	84.50	yes
Breast tumor 41	GSE16987	GPL6104	GSM425180	1699538090_B	Tissue	7.6	43	1	IDC	1.5	3	4(13)	(+)	(-)	(+)	(+)	(-)	1	1	2.00	yes
Breast tumor 42	GSE16987	GPL6104	GSM425181	1699538090_C	Tissue	7.0	69	3	IDC	2.5	2	7(13)	(-)	(-)	(+)	(-)	(-)	1	0	99.83	yes
Breast tumor 43	GSE16987	GPL6104	GSM425182	1699538090_D	Tissue	7.5	42	0.7	IDC	2.9	3	2(27)	(+)	(-)	(+)	(+)	(-)	1	0	95.23	yes
Breast tumor 44	GSE16987	GPL6104	GSM425183	1699538090_E	Tissue	6.6	57	0.4	IDC	4.7	3	7(15)	(+)	(+)	(-)	(-)	(+)	3	0	97.83	yes
Breast tumor 45	GSE16987	GPL6104	GSM425184	1699538090_F	Tissue	7.5	46	0.3	IDC	2.2	3	2(17)	(+)	(+)	(-)	(-)	(+)	3	0	83.40	yes

Sample_title	GEO_series_accession	GEO_platform_accession	GEO_sample_accession	GEO_raw_data/rep	Sample_type/rep	Sample_RIN/rep	Patient_age	Surgical_margin (cm)	Tumor_type	Tumor_size(cm)	Tumor_grade	Positive_LN(total_LN)	LVI	EIC	ER	PR	HER2	CMTC	Recurrence	Recurrence-free_months	Used_in_the_431BC_dataset?
Breast tumor 46	GSE16987	GPL6104	GSM425185	1699538090_G	Tissue	8.4	65	0.04	IDC	1.5	2	1(2)	(+)	(-)	(+)	(+)	(-)	1	0	57.50	yes
Breast tumor 47	GSE16987	GPL6104	GSM425186	1699538091_A	Tissue	8.9	35	0	IDC	6	2	1(18)	(+)	(+)	(+)	(-)	(-)	2	0	79.57	yes
Breast tumor 48	GSE16987	GPL6104	GSM425187	1699538091_B	Tissue	8.2	73	0	IDC	6	1	0(9)	(-)	(-)	(+)	(+)	(-)	1	0	86.83	yes
Breast tumor 49	GSE16987	GPL6104	GSM425188	1699538091_C	Tissue	7.9	44	0.4	IDC	2.65	3	0(3)	(-)	(-)	(-)	(-)	(-)	3	0	76.70	yes
Breast tumor 50	GSE16987	GPL6104	GSM425189	1699538091_D	Tissue	7.0	57	0.48	IDC	1.3	3	2(14)	(+)	(-)	(+)	(-)	(-)	2	0	46.37	yes
Breast tumor 51	GSE16987	GPL6104	GSM425190	1699538091_E	Tissue	7.3	71	1.8	IDC	5	2	1(11)	(+)	(-)	(+)	(+)	(-)	1	0	44.43	yes
Breast tumor 52	GSE16987	GPL6104	GSM425191	1699538091_F	Tissue	6.9	54	0.76	IDC	3.9	3	1(17)	(+)	(-)	(-)	(-)	(+)	3	1	23.67	yes
Breast tumor 53	GSE16987	GPL6104	GSM425192	1855529004_B	FNA	6.6	47	0.2	IDC/Lobular	6	2	1(2)	(-)	(-)	(+)	(+)	(-)	1	0	70.80	yes
Breast tumor 54	GSE16987	GPL6104	GSM425193	1855529004_C/1787466026_F	CBX/FNA	7.9/7.7	54	0.6	IDC	2.5	2	1(22)	(+)	(-)	(+)	(+)	(-)	2	0	69.23	yes
Breast tumor 55	GSE16987	GPL6104	GSM425194	1855529004_D	FNA	9.4	69	0.23	IDC	2.9	2	1(16)	(+)	(-)	(+)	(+)	(-)	1	0	60.40	yes
Breast tumor 56	GSE16987	GPL6104	GSM425195	1855529004_E	FNA	7.5	45	0.35	IDC	1.7	3	0(2)	(+)	(-)	(-)	(-)	(+)	3	0	45.97	yes
Breast tumor 57	GSE16987	GPL6104	GSM425196	1855529010_B	CBX	7	49	0.1	ILC	15	2	5(15)	(-)	(-)	(+)	(+)	(-)	1	0	69.70	yes
Breast tumor 58	GSE16987	GPL6104	GSM425197	1855529010_C	FNA	8.3	59	0.2	IDC	1.6	1	1(17)	(+)	(-)	(+)	(+)	(-)	1	0	59.40	yes
Breast tumor 59	GSE16987	GPL6104	GSM425198	1855529010_D	FNA	8.3	76	1	IDC	2.5	2	0(1)	(+)	(-)	(+)	(-)	(-)	2	0	39.90	yes
Breast tumor 60	GSE16987	GPL6104	GSM425199	1855529010_E	CBX	7	53	0.3	IDC	2.2	3	0(6)	(-)	(-)	(-)	(-)	(-)	3	1	67.03	yes
Breast tumor 61	GSE16987	GPL6104	GSM425200	1855529017_B	FNA	7.4	46	0.6	IDC	2.4	2	0(4)	(-)	(+)	(+)	(+)	(-)	1	0	73.57	yes
Breast tumor 62	GSE16987	GPL6104	GSM425201	1855529017_C	FNA	7.1	73	0.28	IDC	1.7	2	0(2)	(-)	(-)	(+)	(+)	(-)	1	0	36.97	yes
Breast tumor 63	GSE16987	GPL6104	GSM425202	1855529017_D	FNA	7.5	67	2.7	IDC	4	3	3(30)	(-)	(-)	(+)	(-)	(-)	2	0	59.23	yes
Breast tumor 64	GSE16987	GPL6104	GSM425203	1855529017_E	FNA	6.8	45	0.5	IDC	0.9	2	0(5)	(+)	(+)	(+)	(+)	(-)	1	0	66.43	yes
Breast tumor 65	GSE16987	GPL6104	GSM425204	1855529033_C/1787466013_F	FNA/FNA	6.9/6.8	62	0.3	IDC	1.9	3	0(1)	(-)	(-)	(-)	(-)	(-)	3	0	62.67	yes
Breast tumor 66	GSE16987	GPL6104	GSM425205	1855529033_D	FNA	8.1	73	0.5	IDC	1.5	1	1(5)	(-)	(-)	(+)	(+)	(-)	1	0	64.90	yes
Breast tumor 67	GSE16987	GPL6104	GSM425206	1855529033_E	FNA	8.8	51	0.3	IDC	2.2	3	1(17)	(-)	(+)	(+)	(+)	(-)	2	0	52.67	yes
Breast tumor 68	GSE16987	GPL6104	GSM425207	1855529057_B	FNA	6.5	72	1	IDC	1.5	2	1(13)	(-)	(-)	(+)	(+)	(-)	1	0	50.10	yes
Breast tumor 69	GSE16987	GPL6104	GSM425208	1855529057_C	FNA	7.5	58	2.1	ILC	8.8	2	5(49)	(-)	(-)	(+)	(+)	(-)	2	0	59.33	yes
Breast tumor 70	GSE16987	GPL6104	GSM425209	1855529057_D	FNA	9.2	41	0.25	IDC	1.4	2	1(14)	(-)	(-)	(+)	(-)	(-)	2	0	48.23	yes
Breast tumor 71	GSE16987	GPL6104	GSM425210	1855529057_E	FNA	7.1	55	0.5	ILC	16.1	2	0(23)	(-)	(-)	(+)	(-)	(-)	1	0	57.23	yes
Breast tumor 72	GSE16987	GPL6104	GSM425211	1699538093_A	FNA	8.5	40	0.4	IDC	2	2	3(17)	(+)	(+)	(+)	(+)	(-)	2	0	64.70	yes
Breast tumor 73	GSE16987	GPL6104	GSM425212	1699538093_B	FNA	8.8	60	0.6	IDC	1.3	2	1(23)	(-)	(-)	(+)	(+)	(-)	2	0	63.00	yes
Breast tumor 74	GSE16987	GPL6104	GSM425213	1699538093_C	FNA	9	32	0.4	IDC	2.6	3	1(13)	(+)	(-)	(+)	(-)	(-)	2	0	59.43	yes
Breast tumor 75	GSE16987	GPL6104	GSM425214	1699538093_D/1699538093_E	FNA/FNA	8.4/8.1	65	0.14	IDC	1.8	2	1(17)	(+)	(-)	(+)	(+)	(-)	1	0	60.90	yes
Breast tumor 76	GSE16987	GPL6104	GSM425215	1699538093_F	FNA	8.8	46	0.2	ILC	2.3	2	1(21)	(-)	(-)	(+)	(+)	(-)	1	0	62.13	yes
Breast tumor 77	GSE16987	GPL6104	GSM425216	1699538093_G	FNA	8.8	52	<0.1	IDC	2	3	0(2)	(-)	(+)	(+)	(-)	(-)	2	0	60.07	yes
Breast tumor 78	GSE16987	GPL6104	GSM425217	1699538161_A	FNA	7.9	58	1.2	IDC	3	3	2(18)	(-)	(-)	(+)	(+)	(-)	3	0	1.80	yes
Breast tumor 79	GSE16987	GPL6104	GSM425218	1699538161_B	FNA	7.4	58	0.26	IDC	0.8	1	0(1)	(-)	(-)	(+)	(+)	(-)	1	0	64.90	yes
Breast tumor 80	GSE16987	GPL6104	GSM425219	1699538161_C/1787466013_G	FNA/FNA	8.7/NA	58	0.13	IDC	0.3	2	1(5)	(-)	(-)	(+)	(-)	(-)	2	0	62.57	yes
Breast tumor 81	GSE16987	GPL6104	GSM425220	1699538161_D	FNA	8.5	50	0.9	DCIS	15	3	0(5)	(-)	NA	(-)	(-)	(+)	NA	0	7.27	no
Breast tumor 82	GSE16987	GPL6104	GSM425221	1699538161_E	FNA	7.3	36	0.1	IDC	3.4	3	0(3)	(-)	(-)	(-)	(-)	(-)	3	0	62.93	yes
Breast tumor 83	GSE16987	GPL6104	GSM425222	1699538161_F	FNA	8.6	76	2.1	IDC	2.7	3	2(18)	(+)	(-)	(+)	(+)	(-)	2	0	65.33	yes
Breast tumor 84	GSE16987	GPL6104	GSM425223	1699538161_G	FNA	8.5	51	0.2	IDC	2.7	3	1(11)	(-)	(-)	(+)	(+)	(-)	2	0	2.63	yes
Breast tumor 85	GSE16987	GPL6104	GSM425224	1787466013_B	FNA	9.4	47	0.7	IDC	2.8	3	1(2)	(-)	(+)	(+)	(+)	(-)	2	0	3.70	yes
Breast tumor 86	GSE16987	GPL6104	GSM425225	1787466013_C	FNA	9.2	60	0.5	IDC	1.5	2	0(2)	(+)	(+)	(+)	(-)	(-)	2	0	50.10	yes
Breast tumor 87	GSE16987	GPL6104	GSM425226	1787466013_D	FNA	9.2	68	0.15	IDC	2.4	3	0(3)	(-)	(-)	(-)	(-)	(+)	3	0	24.67	yes
Breast tumor 88	GSE16987	GPL6104	GSM425227	1787466013_E	FNA	9.2	59	0.25	IDC	2.7	3	0(1)	(-)	(-)	(-)	(-)	(+)	3	0	30.80	yes
Breast tumor 89	GSE16987	GPL6104	GSM425228	1787466026_A/1888849036_D	FNA/FNA	6.3/4.8	71	0.1	IDC	2.4	2	0(5)	(-)	(+)	(-)	(-)	(+)	3	0	33.33	yes
Breast tumor 90	GSE16987	GPL6104	GSM425229	1787466026_B	CBX	3.5	54	0.1	IDC	1.6	2	0(2)	(-)	(-)	(+)	(+)	(-)	NA	NA	NA	no
Breast tumor 91	GSE16987	GPL6104	GSM425230	1787466026_C	CBX	4.1	60	NA	Benign	NA	NA	NA	NA	NA	NA	NA	NA	NA	NA	NA	no
Breast tumor 92	GSE16987	GPL6104	GSM425231	1787466026_D	CBX	2.7	42	NA	Benign	NA	NA	0(0)	NA	NA	NA	NA	NA	NA	NA	NA	no

Sample_title	GEO_series_accession	GEO_platform_accession	GEO_sample_accession	GEO_raw_data/rep	Sample_type/rep	Sample_RIN/rep	Patient_age	Surgical_margin (cm)	Tumor_type	Tumor_size(cm)	Tumor_grade	Positive_LN(total_LN)	LVI	EIC	ER	PR	HER2	CMTC	Recurrence	Recurrence-free_months	Used_in_the_431BC_dataset?
Breast tumor 93	GSE16987	GPL6104	GSM425232	1787466026_E	CBX	NA	49	NA	Benign	NA	NA	NA	NA	NA	NA	NA	NA	NA	NA	NA	no
Breast tumor 94	GSE16987	GPL6104	GSM425233	1787466026_G	CBX	7.2	57	0.2	IDC	1.5	1	0(3)	(-)	(-)	(+)	(-)	(-)	1	0	61.90	yes
Breast tumor 95	GSE16987	GPL6104	GSM425234	1787466037_A	FNA	6.8	60	NA	Benign	NA	NA	0(4)	NA	NA	NA	NA	NA	NA	0	24.17	no
Breast tumor 96	GSE16987	GPL6104	GSM425235	1787466037_B	FNA	8.6	53	0	ILC	0.8	2	0(5)	(+)	(-)	(+)	(+)	(-)	2	0	40.00	yes
Breast tumor 97	GSE16987	GPL6104	GSM425236	1787466037_C	FNA	9.3	35	0.075	IDC	5.9	3	6(19)	(+)	(+)	(+)	(+)	(-)	2	0	15.97	yes
Breast tumor 98	GSE16987	GPL6104	GSM425237	1787466037_D	FNA	6.9	59	0.3	IDC	1	3	0(2)	(-)	(+)	(+)	(-)	(+)	1	0	51.73	yes
Breast tumor 99	GSE16987	GPL6104	GSM425238	1787466037_E	FNA	8.8	47	0.15	IDC	1.9	2	1(19)	(-)	(-)	(+)	(+)	(-)	2	0	62.10	yes
Breast tumor 100	GSE16987	GPL6104	GSM425239	1787466037_F	FNA	9.0	68	0.3	IDC	1.4	2	0(3)	(-)	(-)	(-)	(-)	(-)	3	0	57.43	yes
Breast tumor 101	GSE16987	GPL6104	GSM425240	1787466037_G	FNA	9.5	35	0.07	IDC	2.6	2	2(5)	(+)	(-)	(-)	(-)	(+)	3	0	38.27	yes
Breast tumor 102	GSE16987	GPL6104	GSM425241	1787466038_A	FNA	9.2	55	1.05	IDC	2.9	3	0(3)	(+)	(-)	(-)	(-)	(+)	3	0	15.80	yes
Breast tumor 103	GSE16987	GPL6104	GSM425242	1787466038_B	FNA	9.0	75	1.1	IDC	2.3	3	1(4)	(-)	(-)	(-)	(-)	(+)	3	0	57.43	yes
Breast tumor 104	GSE16987	GPL6104	GSM425243	1787466038_C	FNA	7.4	47	0.16	IDC	2.5	3	3(24)	(-)	(-)	(+)	(+)	(-)	2	1	28.37	yes
Breast tumor 105	GSE16987	GPL6104	GSM425244	1787466038_D	FNA	9.3	64	0.9	IDC	3	3	2(38)	(+)	(+)	(+)	(+)	(-)	2	0	63.20	yes
Breast tumor 106	GSE16987	GPL6104	GSM425245	1787466038_E	CBX	8.1	66	0.9	IDC	2.3	2	1(19)	(+)	(-)	(+)	(+)	(+)	1	0	53.00	yes
Breast tumor 107	GSE16987	GPL6104	GSM425246	1787466038_F	FNA	6.5	63	0.8	IDC	1.6	3	0(5)	(-)	(-)	(-)	(-)	(-)	3	0	63.77	yes
Breast tumor 108	GSE16987	GPL6104	GSM425247	1787466038_G	FNA	6.7	83	0.45	DCIS	1.5	2	0(0)	(-)	NA	NA	NA	NA	NA	0	18.50	no
Breast tumor 109	GSE16987	GPL6104	GSM425248	1787466039_A	FNA	6.7	53	1.3	IDC	3.5	3	2(19)	(-)	(+)	(-)	(-)	(+)	3	0	47.63	yes
Breast tumor 110	GSE16987	GPL6104	GSM425249	1787466039_B	FNA	9.6	61	0.3	IDC	2.2	3	0(2)	(-)	(-)	(+)	(+)	(-)	2	0	59.97	yes
Breast tumor 111	GSE16987	GPL6104	GSM425250	1787466039_C	FNA	7.3	69	0.4	IDC	1.3	2	3(10)	(-)	(-)	(+)	(+)	(-)	2	0	57.47	yes
Breast tumor 112	GSE16987	GPL6104	GSM425251	1787466039_D	FNA	5.6	66	0.4	ILC	2.1	2	2(18)	(-)	(-)	(+)	(+)	(-)	1	0	54.77	yes
Breast tumor 113	GSE16987	GPL6104	GSM425252	1787466039_E	FNA	7.4	50	0.9	IDC/ILC	2.6	3	0(3)	(+)	(-)	(-)	(-)	(+)	3	0	50.80	yes
Breast tumor 114	GSE16987	GPL6104	GSM425253	1787466039_F	FNA	9.1	62	0.9	IDC	2.5	3	2(12)	(+)	(-)	(+)	(+)	(-)	2	0	27.97	yes
Breast tumor 115	GSE16987	GPL6104	GSM425254	1787466039_G	FNA	9.0	45	0.3	IDC	2.2	3	2(17)	(+)	(+)	(+)	(+)	(+)	2	0	60.70	yes
Breast tumor 116	GSE16987	GPL6104	GSM425255	1888849028_A	FNA	6.4	85	0.5	IDC	1.5	2	0(2)	(-)	(-)	(+)	(+)	(-)	1	0	52.03	yes
Breast tumor 117	GSE16987	GPL6104	GSM425256	1888849028_B	FNA	8.3	38	0.15	IDC	4.5	3	2(10)	(-)	(+)	(-)	(-)	(+)	3	0	34.03	yes
Breast tumor 118	GSE16987	GPL6104	GSM425257	1888849028_C	FNA	7.0	81	1.3	DCIS/IDC	0.11	NA	0(3)	(-)	(-)	(+)	(+)	(-)	NA	0	17.90	no
Breast tumor 119	GSE16987	GPL6104	GSM425258	1888849028_D/1888849036_G	FNA/FNA	5.8/5.4	77	0.42	ILC	2.4	2	0(2)	(-)	(-)	(+)	(+)	(-)	1	0	52.57	yes
Breast tumor 120	GSE16987	GPL6104	GSM425259	1888849028_E	FNA	6.5	43	0.2	DCIS	7	3	0(4)	(-)	(-)	NA	NA	NA	NA	0	16.73	no
Breast tumor 121	GSE16987	GPL6104	GSM425260	1888849028_F	FNA	7.2	53	0.24	IDC	2.6	3	1(15)	(+)	(-)	(+)	(-)	(-)	2	0	23.20	yes
Breast tumor 122	GSE16987	GPL6104	GSM425261	1888849028_G	FNA	6.1	34	0.05	IDC	2.5	2	0(3)	(-)	(-)	(+)	(-)	(-)	2	0	58.17	yes
Breast tumor 123	GSE16987	GPL6104	GSM425262	1888849036_A	FNA	7.3	67	0.05	IDC	2.5	3	0(2)	(-)	(-)	(+)	(+)	(-)	2	0	51.57	yes
Breast tumor 124	GSE16987	GPL6104	GSM425263	1888849036_B	FNA	7.9	41	1	IDC	1.1	3	0(2)	(-)	(-)	(-)	(-)	(-)	3	0	54.20	yes
Breast tumor 125	GSE16987	GPL6104	GSM425264	1888849036_C	FNA	8.2	60	0.1	IDC	3	3	0(2)	(-)	(-)	(-)	(-)	(+)	3	1	21.50	yes
Breast tumor 126	GSE16987	GPL6104	GSM425265	1888849036_E	FNA	4.5	70	1.5	ILC	11	2	0(20)	(-)	(-)	(+)	(-)	(-)	NA	NA	NA	no
Breast tumor 127	GSE16987	GPL6104	GSM425266	1888849036_F	FNA	7.7	59	0.5	IDC	2.8	3	0(4)	(-)	(-)	(+)	(+)	(-)	2	0	56.47	yes
Breast tumor 128	GSE16987	GPL6104	GSM425267	4098316007_A	FNA	7.8	65	0.5	IDC	2.4	3	0(4)	(-)	(-)	(+)	(-)	(-)	2	0	59.80	yes
Breast tumor 129	GSE16987	GPL6104	GSM425268	4098316007_B	FNA	8.7	73	0.34	IDC	2	2	0(1)	(-)	(-)	(+)	(+)	(-)	2	0	59.37	yes
Breast tumor 130	GSE16987	GPL6104	GSM425269	4098316007_C	FNA	6.7	50	0.1	IDC	1.1	1	0(2)	(-)	(+)	(+)	(-)	(-)	1	0	31.57	yes
Breast tumor 131	GSE16987	GPL6104	GSM425270	4098316007_D	FNA	8.5	46	0.03	IDC	1.8	3	5(35)	(+)	(-)	(-)	(-)	(-)	3	0	41.60	yes
Breast tumor 132	GSE16987	GPL6104	GSM425271	4098316007_E	FNA	9.4	65	0.04	IDC	2.5	3	2(14)	(+)	(-)	(+)	(+)	(-)	2	0	11.00	yes
Breast tumor 133	GSE16987	GPL6104	GSM425272	4098316007_F	FNA	6.7	59	0	IDC	10.8	3	0(0)	(+)	(-)	(+)	(-)	(-)	2	0	50.90	yes
Breast tumor 134	GSE16987	GPL6104	GSM425273	4098316007_G	FNA	6.8	55	0.28	IDC	3	3	0(6)	(-)	(-)	(-)	(-)	(-)	3	0	26.40	yes
Breast tumor 135	GSE16987	GPL6104	GSM425274	4098316013_A	FNA	5.7	61	0.23	IDC	2	2	16(24)	(-)	(-)	(+)	(+)	(-)	2	0	49.73	yes
Breast tumor 136	GSE16987	GPL6104	GSM425275	4098316013_B	FNA	8.3	48	0.45	IMC	3.2	2	0(7)	(-)	(+)	(+)	(+)	(-)	2	0	57.07	yes
Breast tumor 137	GSE16987	GPL6104	GSM425276	4098316013_C	FNA	6.9	48	0.3	IDC	6	3	12(20)	(+)	(-)	(-)	(-)	(+)	3	1	15.53	yes
Breast tumor 138	GSE16987	GPL6104	GSM425277	4098316013_D	FNA	7.2	49	0.5	IDC	1.6	2	1(20)	(-)	(-)	(+)	(+)	(-)	2	0	58.37	yes
Breast tumor 139	GSE16987	GPL6104	GSM425278	4098316013_E	FNA	7.8	75	NA	ILC	7	3	3(17)	(+)	(-)	(+)	(+)	(-)	2	0	29.60	yes

Sample_title	GEO_series_accession	GEO_platform_accession	GEO_sample_accession	GEO_raw_data/rep	Sample_type/rep	Sample_RIN/rep	Patient_age	Surgical_margin (cm)	Tumor_type	Tumor_size(cm)	Tumor_grade	Positive_LN(total_LN)	LVI	EIC	ER	PR	HER2	CMTC	Recurrence	Recurrence-free_months	Used_in_the_431BC_dataset?
Breast tumor 140	GSE16987	GPL6104	GSM425279	4098316013_F	FNA	8.8	42	0.3	IDC	2.4	2	1(15)	(-)	(-)	(+)	(-)	(-)	2	0	49.50	yes
Breast tumor 141	GSE16987	GPL6104	GSM425280	4098316013_G	FNA	8.0	52	0	IDC	3	3	1(3)	(+)	(-)	(+)	(-)	(+)	2	0	49.60	yes
Breast tumor 142	GSE16987	GPL6104	GSM425281	1888849005_B	FNA	7.3	54	0.06	IDC	2.1	3	0(3)	(-)	(-)	(+)	(+)	(-)	2	0	45.50	yes
Breast tumor 143	GSE16987	GPL6104	GSM425282	1888849005_C	FNA	8.4	53	0.32	IDC	3.4	3	0(3)	(+)	(-)	(-)	(-)	(+)	3	1	13.97	yes
Breast tumor 144	GSE16987	GPL6104	GSM425283	1888849005_D	FNA	7.5	53	0.21	IDC	3.6	3	14(21)	(-)	(-)	(-)	(-)	(+)	3	1	22.57	yes
Breast tumor 145	GSE16987	GPL6104	GSM425284	1888849005_E	FNA	7.2	48	0.3	IMC	7.5	2	14(19)	(-)	(-)	(+)	(+)	(-)	2	0	58.33	yes
Breast tumor 146	GSE16987	GPL6104	GSM425285	1888849005_F	FNA	6.2	48	0.23	IDC	1.7	2	5(14)	(+)	(-)	(+)	(+)	(-)	1	0	50.93	yes
Breast tumor 147	GSE16987	GPL6104	GSM425286	1888849005_G	FNA	7.3	57	0.4	IDC	1.2	3	0(2)	(-)	(-)	(+)	(+)	(-)	2	0	54.20	yes
Breast tumor 148	GSE16987	GPL6104	GSM425287	1888849006_A	FNA	7.9	51	0.1	IDC	4	3	2(21)	(+)	(-)	(+)	(+)	(-)	2	0	53.87	yes
Breast tumor 149	GSE16987	GPL6104	GSM425288	1888849006_B	FNA	8.6	30	0	IDC	2.4	3	2(18)	(+)	(-)	(+)	(-)	(-)	2	0	54.20	yes
Breast tumor 150	GSE16987	GPL6104	GSM425289	1888849006_C	FNA	8.0	60	NA	IDC	1.6	1	0(1)	(-)	NA	(+)	(-)	(-)	2	0	41.13	yes
Breast tumor 151	GSE16987	GPL6104	GSM425290	1888849006_D	FNA	7.1	67	0.11	IDC	1.2	2	0(5)	(+)	(-)	(+)	(-)	(-)	1	0	18.77	yes
Breast tumor 152	GSE16987	GPL6104	GSM425291	1888849006_E	FNA	7.5	72	0.3	IDC	2.1	2	0(3)	(+)	(-)	(+)	(+)	(-)	2	0	48.57	yes
Breast tumor 153	GSE16987	GPL6104	GSM425292	1888849006_F	FNA	7.8	43	0.4	IDC	2.3	2	0(2)	(-)	(-)	(-)	(-)	(-)	3	0	51.80	yes
Breast tumor 154	GSE16987	GPL6104	GSM425293	1888849006_G	FNA	8.3	66	0.8	IDC	1.9	3	0(4)	(-)	(-)	(+)	(-)	(-)	2	0	57.20	yes
Breast tumor 155	GSE16987	GPL6104	GSM425294	4098316046_A	FNA	5.6	69	3.5	IDC	1.8	3	0(1)	(-)	NA	(-)	(-)	(-)	3	0	54.53	yes
Breast tumor 156	GSE16987	GPL6104	GSM425295	4098316046_B	FNA	8.7	52	0.8	IDC	2.1	1	0(2)	(-)	(-)	(+)	(+)	(-)	1	0	47.60	yes
Breast tumor 157	GSE16987	GPL6104	GSM425296	4098316046_C	FNA	7.9	45	0.005	IDC	3.2	2	4(20)	(+)	(-)	(+)	(-)	(-)	2	0	55.77	yes
Breast tumor 158	GSE16987	GPL6104	GSM425297	4098316046_D	FNA	7.8	78	0.5	IDC	1.4	3	0(1)	(-)	NA	(-)	(-)	(-)	3	0	54.97	yes
Breast tumor 159	GSE16987	GPL6104	GSM425298	4098316046_E	FNA	7.3	58	0.21	IDC	1.4	2	0(3)	(-)	(-)	(+)	(+)	(-)	1	0	52.93	yes
Breast tumor 160	GSE16987	GPL6104	GSM425299	4098316046_F	FNA	8.3	81	0.1	IDC	1.5	2	2(17)	(+)	(-)	(+)	(+)	(-)	2	0	46.23	yes
Breast tumor 161	GSE16987	GPL6104	GSM425300	4098316046_G	FNA	8.3	73	0.2	IDC	0.8	2	0(1)	(+)	(+)	(+)	(+)	(-)	2	0	46.30	yes
Breast tumor 162	GSE45725	GPL6883	GSM1112901	4256290013_C	FNA	6.7	44	0.7	IDC	3.2	3	0(1)	(-)	(-)	(-)	(-)	(+)	3	0	46.90	yes
Breast tumor 163	GSE45725	GPL6883	GSM1112902	4256290013_D	FNA	6.9	72	0.13	IDC	2.2	2	0(5)	(-)	(-)	(-)	(-)	(-)	3	0	17.90	yes
Breast tumor 164	GSE45725	GPL6883	GSM1112903	4256290013_E	FNA	6.8	74	0.09	IDC	2.5	3	0(2)	(+)	(-)	(+)	(+)	(-)	2	0	49.93	yes
Breast tumor 165	GSE45725	GPL6883	GSM1112904	4256290013_F	FNA	7.7	50	NA	Benign	NA	NA	NA	NA	NA	NA	NA	NA	3	NA	NA	no
Breast tumor 166	GSE45725	GPL6883	GSM1112905	4256290013_G	FNA	6.3	78	0.3	IDC	2.2	2	0(2)	(-)	(-)	(+)	(+)	(-)	2	0	0.90	no
Breast tumor 167	GSE45725	GPL6883	GSM1112906	4334518081_A	FNA	6.5	49	0.5	ILC	3.1	2	0(3)	(-)	(-)	(+)	(+)	(-)	1	0	46.33	yes
Breast tumor 168	GSE45725	GPL6883	GSM1112907	4334518081_B	FNA	7.5	56	0.3	IDC	1.2	2	0(1)	(-)	(-)	(+)	(+)	(-)	3	0	47.93	yes
Breast tumor 169	GSE45725	GPL6883	GSM1112908	4334518081_C	FNA	8.8	77	1.3	IDC	2	1	0(3)	(-)	(-)	(+)	(+)	(-)	1	0	34.70	yes
Breast tumor 170	GSE45725	GPL6883	GSM1112909	4334518081_D	FNA	8.3	61	0.225	IDC	2.2	3	2(18)	(+)	(+)	(+)	(-)	(+)	3	0	48.93	yes
Breast tumor 171	GSE45725	GPL6883	GSM1112910	4334518081_E	FNA	7.2	61	0.6	IMC	1.1	2	0(5)	(-)	(-)	(+)	(-)	(-)	1	0	47.67	yes
Breast tumor 172	GSE45725	GPL6883	GSM1112911	4334518081_F	FNA	6.9	56	0.65	IDC	0.7	1	0(2)	(-)	(-)	(+)	(+)	(-)	1	0	45.43	yes
Breast tumor 173	GSE45725	GPL6883	GSM1112912	4334518081_G	FNA	7	61	0.5	IDC	0.5	1	1(2)	(-)	(-)	(+)	(+)	(-)	1	0	42.30	yes
Breast tumor 174	GSE45725	GPL6883	GSM1112913	4334518087_A	FNA	7	79	0.66	ILC	3.6	2	0(4)	(-)	(-)	(+)	(-)	(-)	1	0	46.83	yes
Breast tumor 175	GSE45725	GPL6883	GSM1112914	4334518087_B	FNA	9.2	47	0.1	IDC	2.2	2	1(12)	(+)	(-)	(+)	(+)	(-)	2	0	51.17	yes
Breast tumor 176	GSE45725	GPL6883	GSM1112915	4334518087_C	FNA	6.3	45	1	IDC	6.4	3	16(19)	(+)	(+)	(+)	(+)	(-)	2	0	45.13	yes
Breast tumor 177	GSE45725	GPL6883	GSM1112916	4334518087_D	FNA	7	63	0.33	IDC	2.4	2	0(1)	(-)	(-)	(+)	(+)	(-)	2	0	49.47	yes
Breast tumor 178	GSE45725	GPL6883	GSM1112917	4334518087_E	FNA	8.9	76	0.2	IDC	2.8	3	0(3)	(-)	(-)	(+)	(+)	(-)	3	0	35.27	yes
Breast tumor 179	GSE45725	GPL6883	GSM1112918	4334518087_F	FNA	7.9	43	0	IDC	2.5	3	9(17)	(+)	(-)	(+)	(+)	(-)	2	0	47.37	yes
Breast tumor 180	GSE45725	GPL6883	GSM1112919	4334518087_G	FNA	8.3	42	1	IDC	3.9	3	1(3)	(+)	(-)	(+)	(-)	(-)	3	1	31.03	yes
Breast tumor 181	GSE45725	GPL6883	GSM1112920	4334518100_A	FNA	6.5	56	0.5	IDC	1.2	3	0(3)	(-)	(-)	(+)	(-)	(-)	1	0	48.77	yes
Breast tumor 182	GSE45725	GPL6883	GSM1112921	4334518100_B	FNA	8.6	66	0.8	IDC	2	2	1(2)	(-)	(-)	(+)	(+)	(-)	1	0	32.63	yes
Breast tumor 183	GSE45725	GPL6883	GSM1112922	4334518100_C	FNA	8.5	55	0.14	IDC	1.7	3	0(2)	(-)	(-)	(+)	(+)	(-)	2	0	48.33	yes
Breast tumor 184	GSE45725	GPL6883	GSM1112923	4334518100_D	FNA	7.7	51	1.2	DCIS	1.2	2	NA	NA	NA	NA	NA	NA	3	0	42.97	no
Breast tumor 185	GSE45725	GPL6883	GSM1112924	4334518100_E	FNA	8.3	51	0.3	IDC	1.6	3	1(23)	(+)	(-)	(-)	(-)	(-)	3	0	36.30	yes
Breast tumor 186	GSE45725	GPL6883	GSM1112925	4334518100_F	FNA	6.9	53	0.175	ILC	7.1	2	3(12)	(-)	(-)	(+)	(+)	(-)	1	0	45.70	yes

Sample_title	GEO_series_accession	GEO_platform_accession	GEO_sample_accession	GEO_raw_data/rep	Sample_type/rep	Sample_RIN/rep	Patient_age	Surgical_margin (cm)	Tumor_type	Tumor_size(cm)	Tumor_grade	Positive_LN(total_LN)	LVI	EIC	ER	PR	HER2	CMTC	Recurrence	Recurrence-free_months	Used_in_the_431BC_dataset?
Breast tumor 187	GSE45725	GPL6883	GSM1112926	4334518100_G	FNA	7.7	68	1.5	IDC	0.88	2	0(1)	(-)	(-)	(+)	(+)	(-)	1	0	41.97	yes
Breast tumor 188	GSE45725	GPL6883	GSM1112927	4334518100_H	FNA	7.3	52	0.68	IDC	1.6	2	1(1)	(+)	(-)	(+)	(-)	(-)	2	0	47.43	yes
Breast tumor 189	GSE45725	GPL6883	GSM1112928	4334518108_A	FNA	6	65	0.48	ILC	1.7	2	2(3)	(-)	(-)	(+)	(+)	(-)	1	0	48.23	yes
Breast tumor 190	GSE45725	GPL6883	GSM1112929	4334518108_B	FNA	7.4	43	0.32	IDC	1.6	3	0(6)	(-)	(-)	(-)	(-)	(-)	3	0	48.60	yes
Breast tumor 191	GSE45725	GPL6883	GSM1112930	4334518108_C	FNA	8.5	50	0.8	IDC	1	2	0(2)	(-)	(+)	(+)	(+)	(-)	1	0	47.73	yes
Breast tumor 192	GSE45725	GPL6883	GSM1112931	4334518108_D	FNA	7.8	54	0.2	IDC	3.9	3	3(11)	(+)	(-)	(+)	(+)	(-)	2	1	24.43	yes
Breast tumor 193	GSE45725	GPL6883	GSM1112932	4334518108_E	FNA	8.5	61	0.2	IDC	1.5	2	0(1)	(-)	(-)	(-)	(-)	(+)	3	0	47.80	yes
Breast tumor 194	GSE45725	GPL6883	GSM1112933	4334518108_F	FNA	6.6	58	0.41	IDC	10.6	2	6(24)	(+)	(-)	(+)	(+)	(-)	1	0	47.00	yes
Breast tumor 195	GSE45725	GPL6883	GSM1112934	4334518108_G	FNA	7.8	64	0.76	DCIS	1.6	3	NA	NA	NA	NA	NA	NA	1	0	39.20	no
Breast tumor 196	GSE45725	GPL6883	GSM1112935	4418463155_A	FNA	7.9	74	0.1	IDC	1.8	2	4(25)	(+)	(-)	(+)	(-)	(-)	2	0	46.60	yes
Breast tumor 197	GSE45725	GPL6883	GSM1112936	4418463155_B	FNA	7.6	78	0.1	IDC	1.05	1	0(2)	(-)	(-)	(+)	(+)	(-)	1	0	46.43	yes
Breast tumor 198	GSE45725	GPL6883	GSM1112937	4418463155_C	FNA	7.6	57	0.8	IDC	2.2	1	0(2)	(-)	(-)	(+)	(+)	(-)	3	0	40.80	yes
Breast tumor 199	GSE45725	GPL6883	GSM1112938	4418463155_D	FNA	3.7	55	1.45	IDC	4	3	0(6)	(-)	(-)	(-)	(+)	(-)	3	0	34.90	no
Breast tumor 200	GSE45725	GPL6883	GSM1112939	4418463155_E	FNA	2.7	80	0.7	IDC	2	1	0(0)	(-)	(-)	(+)	(+)	(-)	1	0	38.47	no
Breast tumor 201	GSE45725	GPL6883	GSM1112940	4418463155_F	FNA	8.4	49	0.02	IDC	0.4	2	0(1)	(-)	(+)	(+)	(+)	(-)	1	0	48.30	yes
Breast tumor 202	GSE45725	GPL6883	GSM1112941	4418463155_G	FNA	7.1	42	0.06	IDC	1.1	2	2(3)	(+)	(-)	(+)	(+)	(-)	2	0	47.67	yes
Breast tumor 203	GSE45725	GPL6883	GSM1112942	4418463155_H	FNA	7.8	54	0.56	DCIS/IDC	0.1	NA	0(0)	(-)	NA	(-)	(-)	NA	3	0	47.90	no
Breast tumor 204	GSE45725	GPL6883	GSM1112943	4418463227_A	FNA	6.8	57	0.001	DCIS/IDC	<0.1	NA	NA	NA	NA	NA	NA	NA	3	0	34.37	no
Breast tumor 205	GSE45725	GPL6883	GSM1112944	4418463227_B	FNA	8.9	50	0.5	IDC	2.6	3	3(16)	(+)	(-)	(+)	(-)	(-)	2	0	26.23	yes
Breast tumor 206	GSE45725	GPL6883	GSM1112945	4418463227_C	FNA	8.5	46	0.25	IDC	1.6	1	0(2)	(-)	(+)	(+)	(-)	(-)	2	0	40.87	yes
Breast tumor 207	GSE45725	GPL6883	GSM1112946	4418463227_D	FNA	6.9	54	0.53	IDC	3.6	2	0(11)	(-)	(+)	(-)	(-)	(-)	3	0	46.67	yes
Breast tumor 208	GSE45725	GPL6883	GSM1112947	4418463227_E	FNA	8.6	50	0	IDC	3.7	2	0(2)	(-)	(+)	(+)	(+)	(-)	1	0	37.60	yes
Breast tumor 209	GSE45725	GPL6883	GSM1112948	4418463227_F	FNA	6.6	45	NA	FEA	NA	NA	NA	NA	NA	NA	NA	NA	1	0	38.30	no
Breast tumor 210	GSE45725	GPL6883	GSM1112949	4418463227_G	FNA	6.9	58	0.36	IDC	0.29	2	0(6)	(-)	NA	(+)	(+)	(+)	3	0	45.57	yes
Breast tumor 211	GSE45725	GPL6883	GSM1112950	4418463227_H	FNA	6.9	56	0.7	IDC	1.4	3	0(3)	(-)	(-)	(-)	(-)	(+)	3	0	44.60	yes
Breast tumor 212	GSE45725	GPL6883	GSM1112951	4482545010_B	FNA	6.5	32	0.01	DCIS/IDC	13	2	4(5)	(-)	(-)	(-)	(-)	(+)	3	0	46.37	no
Breast tumor 213	GSE45725	GPL6883	GSM1112952	4482545010_C	FNA	7.2	51	0.6	IDC	4.4	2	2(16)	(-)	(-)	(+)	(+)	(-)	1	0	38.30	yes
Breast tumor 214	GSE45725	GPL6883	GSM1112953	4482545010_D	FNA	8.5	48	1.1	IDC	3.8	3	0(9)	(-)	(-)	(+)	(+)	(+)	2	0	35.27	yes
Breast tumor 215	GSE45725	GPL6883	GSM1112954	4482545010_E	FNA	7	69	0.024	IDC	2.3	3	0(2)	(-)	(-)	(+)	(-)	(+)	3	1	14.50	yes
Breast tumor 216	GSE45725	GPL6883	GSM1112955	4482545010_F	FNA	7.9	43	0.5	IDC	2.5	3	0(5)	(-)	(-)	(+)	(-)	(+)	3	1	25.50	yes
Breast tumor 217	GSE45725	GPL6883	GSM1112956	4482545010_G	FNA	7.8	46	0.4	IDC	1.8	2	0(2)	(-)	(-)	(+)	(+)	(-)	2	0	46.60	yes
Breast tumor 218	GSE45725	GPL6883	GSM1112957	4482545010_H	FNA	7.7	74	0.1	IDC	2.7	2	0(0)	(-)	(-)	(+)	(+)	(-)	2	0	6.60	yes
Breast tumor 219	GSE45725	GPL6883	GSM1112958	4482545111_A	FNA	9.4	55	1.8	IDC	1.9	2	2(15)	(-)	(-)	(+)	(+)	(-)	1	0	1.37	no
Breast tumor 220	GSE45725	GPL6883	GSM1112959	4482545111_B	FNA	8	92	0.1	IDC	3.4	2	4(13)	(+)	(+)	(+)	(+)	(-)	2	1	39.23	yes
Breast tumor 221	GSE45725	GPL6883	GSM1112960	4482545111_C	FNA	7.7	78	0.4	IDC	1.4	2	0(4)	(-)	(-)	(+)	(+)	(-)	1	0	44.43	yes
Breast tumor 222	GSE45725	GPL6883	GSM1112961	4482545111_D	FNA	9	37	0.01	IDC	1.5	3	0(18)	(+)	(+)	(+)	(+)	(+)	2	0	45.30	yes
Breast tumor 223	GSE45725	GPL6883	GSM1112962	4482545111_E	FNA	6.5	73	0.6	IDC	1.6	3	0(2)	(+)	(-)	(-)	(-)	(+)	3	0	46.63	yes
Breast tumor 224	GSE45725	GPL6883	GSM1112963	4482545111_F	FNA	7	49	0.1	IDC	1.7	3	0(6)	(+)	(-)	(+)	(+)	(-)	1	0	45.47	yes
Breast tumor 225	GSE45725	GPL6883	GSM1112964	4482545111_G	FNA	6.6	58	0.71	DCIS/IDC	0.09	NA	1(3)	(-)	(-)	NA	NA	NA	3	0	40.33	no
Breast tumor 226	GSE45725	GPL6883	GSM1112965	4482545111_H	FNA	7.4	64	0.16	IDC	1.5	2	0(4)	(-)	(-)	(+)	(+)	(-)	2	0	16.07	yes
Breast tumor 227	GSE45725	GPL6883	GSM1112966	4801489005_C	FNA	6.4	73	0.52	IDC	1.4	3	0(2)	(+)	(-)	(-)	(-)	(-)	3	1	15.40	yes
Breast tumor 228	GSE45725	GPL6883	GSM1112967	4801489005_D	FNA	5.8	65	0.25	IDC	2.4	3	2(13)	(+)	(-)	(-)	(-)	(-)	3	NA	NA	no
Breast tumor 229	GSE45725	GPL6883	GSM1112968	4801489005_E	FNA	8.7	67	0.61	IDC	3.8	1	0(18)	(+)	(-)	(+)	(+)	(-)	1	0	44.13	yes
Breast tumor 230	GSE45725	GPL6883	GSM1112969	4801489005_F	FNA	9.1	45	0.1	IDC	1.3	3	0(1)	(-)	(-)	(+)	(+)	(-)	3	0	39.07	yes
Breast tumor 231	GSE45725	GPL6883	GSM1112970	4801489005_G	FNA	6.2	49	1.1	DCIS/IDC	0.1	NA	0(4)	(-)	(+)	(+)	(-)	NA	3	0	39.50	no
Breast tumor 232	GSE45725	GPL6883	GSM1112971	4801489005_H	FNA	8.2	65	0.2	IDC	0.7	2	0(3)	(-)	(+)	(+)	(+)	(-)	3	0	40.40	yes
Breast tumor 233	GSE45725	GPL6883	GSM1112972	4801489010_A	FNA	9.3	41	0.01	IDC	1.6	3	0(3)	(-)	(-)	(+)	(+)	(+)	2	0	43.33	yes

Sample_title	GEO_series_accession	GEO_platform_accession	GEO_sample_accession	GEO_raw_data/rep	Sample_type/rep	Sample_RIN/rep	Patient_age	Surgical_margin (cm)	Tumor_type	Tumor_size(cm)	Tumor_grade	Positive_LN(total_LN)	LVI	EIC	ER	PR	HER2	CMTC	Recurrence	Recurrence-free_months	Used_in_the_431BC_dataset?
Breast tumor 234	GSE45725	GPL6883	GSM1112973	4801489010_B	FNA	8.6	53	0.8	IDC	2.3	3	0(4)	(+)	(-)	(+)	(+)	(-)	1	0	40.23	yes
Breast tumor 235	GSE45725	GPL6883	GSM1112974	4801489010_C	FNA	5.9	47	0.05	IDC	1.2	1	0(4)	(-)	(-)	(+)	(+)	(-)	1	0	40.20	yes
Breast tumor 236	GSE45725	GPL6883	GSM1112975	4801489010_D	FNA	7.2	45	1.1	IDC	1.6	2	0(5)	(-)	(-)	(+)	(+)	(-)	1	1	35.00	yes
Breast tumor 237	GSE45725	GPL6883	GSM1112976	4801489010_E	FNA	7.5	55	0.5	IDC	4.5	3	3(4)	(+)	(+)	(+)	(+)	(-)	1	0	36.77	yes
Breast tumor 238	GSE45725	GPL6883	GSM1112977	4801489010_F	FNA	4.7	34	NA	DCIS	NA	NA	0(7)	NA	NA	NA	NA	NA	2	0	42.30	no
Breast tumor 239	GSE45725	GPL6883	GSM1112978	4801489010_G	FNA	6.6	62	0.9	IDC	1.6	3	1(21)	(+)	(+)	(-)	(-)	(-)	3	1	34.17	yes
Breast tumor 240	GSE45725	GPL6883	GSM1112979	4801489010_H	FNA	7.4	39	NA	DCIS	NA	NA	0(2)	NA	NA	NA	NA	NA	1	0	37.10	no
Breast tumor 241	GSE45725	GPL6883	GSM1112980	4801489011_A	FNA	7.2	44	0.1	IDC	1.9	2	0(1)	(+)	(+)	(+)	(+)	(+)	2	0	41.30	yes
Breast tumor 242	GSE45725	GPL6883	GSM1112981	4801489011_B	FNA	7.2	68	2.1	IDC	2	2	4(17)	(-)	(-)	(+)	(+)	(-)	2	0	30.73	yes
Breast tumor 243	GSE45725	GPL6883	GSM1112982	4801489011_C	FNA	7.7	31	0.01	IDC	3.1	3	2(14)	(+)	(-)	(+)	(+)	(-)	2	0	43.00	yes
Breast tumor 244	GSE45725	GPL6883	GSM1112983	4801489011_D	FNA	7.8	42	0.2	IDC	2.6	2	0(3)	(-)	(-)	(+)	(+)	(-)	1	0	35.40	yes
Breast tumor 245	GSE45725	GPL6883	GSM1112984	4801489011_E	FNA	7.3	66	0.34	IDC	1.2	2	0(3)	(+)	(-)	(+)	(+)	(-)	1	0	39.40	yes
Breast tumor 246	GSE45725	GPL6883	GSM1112985	4801489011_F	FNA	7	49	1.5	IDC	1.9	2	0(3)	(-)	(-)	(+)	(+)	(-)	1	0	40.87	yes
Breast tumor 247	GSE45725	GPL6883	GSM1112986	4801489011_G	FNA	5.6	76	0.8	IDC	2.3	3	2(20)	(+)	(-)	(-)	(-)	(+)	3	0	35.37	yes
Breast tumor 248	GSE45725	GPL6883	GSM1112987	4801489011_H	FNA	7.5	50	1.3	IDC	0.8	2	0(4)	(-)	(+)	(+)	(+)	(-)	1	0	12.57	yes
Breast tumor 249	GSE45725	GPL6883	GSM1112988	4801489012_A	FNA	8.8	71	0.9	IDC	1.6	2	0(7)	(-)	(-)	(+)	(+)	(-)	2	0	1.47	no
Breast tumor 250	GSE45725	GPL6883	GSM1112989	4801489012_B	FNA	7.2	49	0.12	IDC	1.8	2	0(5)	(-)	(-)	(+)	(+)	(-)	1	0	40.83	yes
Breast tumor 251	GSE45725	GPL6883	GSM1112990	4801489012_C	FNA	8.7	48	0.7	IDC	0.9	1	0(3)	(-)	(-)	(+)	(+)	(-)	1	0	33.53	yes
Breast tumor 252	GSE45725	GPL6883	GSM1112991	4801489012_D	FNA	6.3	65	0.9	IDC	0.8	2	0(1)	(-)	(-)	(+)	(-)	(-)	1	0	40.17	yes
Breast tumor 253	GSE45725	GPL6883	GSM1112992	4801489012_E	FNA	5.6	47	0.05	IDC	1.5	3	0(4)	(-)	(-)	(-)	(-)	(-)	3	0	41.37	yes
Breast tumor 254	GSE45725	GPL6883	GSM1112993	4801489012_F	FNA	9.1	78	0.06	IDC	1.7	2	0(1)	(-)	(-)	(+)	(+)	(-)	1	0	32.93	yes
Breast tumor 255	GSE45725	GPL6883	GSM1112994	4801489012_G	FNA	8.4	71	0.7	IDC	1.9	3	0(3)	(-)	(-)	(+)	(+)	(-)	2	0	40.33	yes
Breast tumor 256	GSE45725	GPL6883	GSM1112995	4801489012_H	FNA	6.1	71	1	IDC	1.2	3	0(2)	(-)	(-)	(+)	(-)	(-)	1	0	42.10	yes
Breast tumor 257	GSE45725	GPL6883	GSM1112996	4801489038_A	FNA	7.2	55	1.6	IDC	1.7	3	14(21)	(+)	(-)	(+)	(-)	(+)	3	0	41.23	yes
Breast tumor 258	GSE45725	GPL6883	GSM1112997	4801489038_B	FNA	NA	NA	0.6	IDC	1.9	1	0(2)	(-)	(-)	(+)	(+)	(-)	1	0	41.33	no
Breast tumor 259	GSE45725	GPL6883	GSM1112998	4801489038_C	FNA	7.1	56	0.3	IDC	3.7	2	0(1)	(+)	(-)	(+)	(-)	(-)	2	0	38.87	yes
Breast tumor 260	GSE45725	GPL6883	GSM1112999	4801489038_D	FNA	NA	85	0.6	IDC	3.4	2	0(1)	(-)	(+)	(+)	(+)	(-)	3	0	31.67	no
Breast tumor 261	GSE45725	GPL6883	GSM1113000	4801489038_E	FNA	7.5	82	0.7	IDC	3.2	1	0(0)	(-)	(+)	(+)	(+)	(-)	2	0	41.47	yes
Breast tumor 262	GSE45725	GPL6883	GSM1113001	4801489038_F	FNA	8	54	1.1	IDC	2.9	3	0(12)	(+)	(-)	(-)	(-)	(+)	3	0	40.40	yes
Breast tumor 263	GSE45725	GPL6883	GSM1113002	4801489038_G	FNA	7.7	39	0.2	IDC	2.4	3	0(2)	(-)	(-)	(-)	(-)	(-)	3	1	14.10	yes
Breast tumor 264	GSE45725	GPL6883	GSM1113003	4801489038_H	FNA	8.9	57	0.08	IDC	2.9	2	0(4)	(+)	(-)	(-)	(-)	(-)	3	0	37.40	yes
Breast tumor 265	GSE45725	GPL6883	GSM1113004	4801489039_A	FNA	7.2	45	0.1	IDC	2.4	3	0(2)	(-)	(-)	(+)	(+)	(+)	2	0	39.27	yes
Breast tumor 266	GSE45725	GPL6883	GSM1113005	4801489039_B	FNA	6.5	80	0.5	IDC	4.2	2	10(14)	(+)	(-)	(+)	(-)	(+)	3	0	25.47	yes
Breast tumor 267	GSE45725	GPL6883	GSM1113006	4801489039_C	FNA	8.8	64	0.8	IDC	2.3	3	5(21)	(+)	(-)	(-)	(-)	(+)	3	0	40.83	yes
Breast tumor 268	GSE45725	GPL6883	GSM1113007	4801489039_D	FNA	9.1	39	0.12	IDC	1.2	3	0(15)	(+)	(-)	(+)	(+)	(-)	2	0	39.70	yes
Breast tumor 269	GSE45725	GPL6883	GSM1113008	4801489039_E	FNA	8.8	55	0.04	IDC	2	3	31(39)	(+)	(-)	(+)	(+)	(-)	2	0	38.73	yes
Breast tumor 270	GSE45725	GPL6883	GSM1113009	4801489039_F	FNA	9	53	0.64	IDC	2.7	3	1(17)	(+)	(-)	(-)	(-)	(-)	3	0	39.50	yes
Breast tumor 271	GSE45725	GPL6883	GSM1113010	4801489039_G	FNA	9.3	45	0.24	IDC	2.7	2	0(1)	(-)	(-)	(+)	(+)	(-)	1	0	38.50	yes
Breast tumor 272	GSE45725	GPL6883	GSM1113011	4801489039_H	FNA	7.3	67	0.2	IDC	3.2	3	1(24)	(+)	(-)	(+)	(+)	(-)	2	1	12.40	yes
Breast tumor 273	GSE45725	GPL6883	GSM1113012	4849554001_A	FNA	8.5	74	0.4	IDC	1.6	1	1(3)	(-)	(-)	(+)	(+)	(-)	1	0	38.73	yes
Breast tumor 274	GSE45725	GPL6883	GSM1113013	4849554001_B	FNA	8.8	63	0.3	IDC	1.8	2	0(2)	(-)	(-)	(+)	(+)	(-)	1	0	35.00	yes
Breast tumor 275	GSE45725	GPL6883	GSM1113014	4849554001_C	FNA	8.6	71	0.4	IDC	0.8	2	0(2)	(-)	(-)	(+)	(+)	(-)	1	0	32.13	yes
Breast tumor 276	GSE45725	GPL6883	GSM1113015	4849554001_D	FNA	7.3	60	0.3	IDC	1.6	3	0(5)	(+)	(-)	(-)	(-)	(-)	3	0	38.23	yes
Breast tumor 277	GSE45725	GPL6883	GSM1113016	4849554001_E	FNA	8.8	78	0.6	IDC	4.1	3	0(3)	(+)	(-)	(-)	(-)	(-)	3	0	24.47	yes
Breast tumor 278	GSE45725	GPL6883	GSM1113017	4849554001_G	FNA	8.4	50	0.6	IDC	3.3	3	5(17)	(+)	(-)	(+)	(+)	(-)	1	0	38.57	yes
Breast tumor 279	GSE45725	GPL6883	GSM1113018	4849554001_H	FNA	7.2	80	0.07	ILC	3.2	1	0(5)	(-)	(-)	(+)	(+)	(-)	2	0	24.77	yes
Breast tumor 280	GSE45725	GPL6883	GSM1113019	4849554002_A	FNA	8.3	75	0.9	IDC	0.8	3	0(3)	(-)	(-)	(+)	(+)	(-)	1	0	36.40	yes

Sample_title	GEO_series_accession	GEO_platform_accession	GEO_sample_accession	GEO_raw_data/rep	Sample_type/rep	Sample_RIN/rep	Patient_age	Surgical_margin (cm)	Tumor_type	Tumor_size(cm)	Tumor_grade	Positive_LN(total_LN)	LVI	EIC	ER	PR	HER2	CMTC	Recurrence	Recurrence-free_months	Used_in_the_431BC_dataset?
Breast tumor 281	GSE45725	GPL6883	GSM1113020	4849554002_B	FNA	9.3	83	0.6	IDC	1.5	3	0(0)	(-)	(-)	(+)	(-)	(-)	3	1	19.63	yes
Breast tumor 282	GSE45725	GPL6883	GSM1113021	4849554002_C	FNA	7.5	59	0	IDC	1.7	3	0(2)	(+)	(-)	(+)	(+)	(-)	1	0	7.80	yes
Breast tumor 283	GSE45725	GPL6883	GSM1113022	4849554002_D	FNA	7.6	67	0.04	ILC	1.8	3	0(5)	(+)	(-)	(+)	(+)	(-)	2	0	34.70	yes
Breast tumor 284	GSE45725	GPL6883	GSM1113023	4849554002_E	FNA	8.2	59	0.5	IDC	2.1	3	0(1)	(-)	(-)	(-)	(-)	(+)	3	0	38.33	yes
Breast tumor 285	GSE45725	GPL6883	GSM1113024	4849554002_F	FNA	7.7	54	1.3	IDC	3.6	3	9(14)	(-)	(+)	(+)	(-)	(-)	3	0	36.90	yes
Breast tumor 286	GSE45725	GPL6883	GSM1113025	4849554002_G	FNA	9.1	73	0	IDC	1.4	2	0(2)	(-)	(-)	(+)	(+)	(-)	1	0	38.60	yes
Breast tumor 287	GSE45725	GPL6883	GSM1113026	4849554002_H	FNA	8.2	89	0.26	IMC	1.4	3	0(1)	(+)	(-)	(+)	(+)	(-)	2	0	35.70	yes
Breast tumor 288	GSE45725	GPL6883	GSM1113027	4849554003_A	FNA	8.8	44	0.28	IDC	2.5	3	0(3)	(+)	(-)	(+)	(+)	(+)	2	0	38.57	yes
Breast tumor 289	GSE45725	GPL6883	GSM1113028	4849554003_B	FNA	9.6	62	0.59	IDC	0.8	2	0(2)	(-)	(+)	(+)	(+)	(-)	2	0	37.73	yes
Breast tumor 290	GSE45725	GPL6883	GSM1113029	4849554003_C	FNA	6.6	77	0.07	IDC	4	3	3(17)	(+)	(-)	(+)	(+)	(+)	2	0	6.30	yes
Breast tumor 291	GSE45725	GPL6883	GSM1113030	4849554003_D	FNA	8	36	0.6	IDC	2.4	3	0(4)	(+)	(-)	(-)	(-)	(+)	3	0	38.30	yes
Breast tumor 292	GSE45725	GPL6883	GSM1113031	4849554003_E	FNA	7.5	43	0	IDC	1.3	2	0(4)	(-)	(-)	(+)	(+)	(-)	2	0	32.13	yes
Breast tumor 293	GSE45725	GPL6883	GSM1113032	4849554003_F	FNA	8.7	75	1.1	IDC	2.9	3	0(6)	(-)	(-)	(+)	(+)	(-)	2	NA	NA	no
Breast tumor 294	GSE45725	GPL6883	GSM1113033	4849554003_G	FNA	8.7	44	0	IDC	3.5	1	4(26)	(-)	(-)	(+)	(+)	(-)	1	0	36.30	yes
Breast tumor 295	GSE45725	GPL6883	GSM1113034	4849554003_H	FNA	7.7	51	1.1	IDC	2.2	3	1(24)	(+)	(-)	(+)	(+)	(-)	2	0	35.47	yes
Breast tumor 296	GSE45725	GPL6883	GSM1113035	4849554043_A	FNA	6.2	49	0.6	IMC	2	2	0(1)	(-)	(-)	(+)	(+)	(-)	2	0	38.57	yes
Breast tumor 297	GSE45725	GPL6883	GSM1113036	4849554043_C	FNA	8.9	69	1.3	IDC	3	3	0(5)	(+)	(-)	(+)	(-)	(+)	2	0	15.53	yes
Breast tumor 298	GSE45725	GPL6883	GSM1113037	4849554043_D	FNA	9.5	57	0.16	IDC	2.2	3	0(2)	(-)	(-)	(+)	(+)	(-)	2	0	33.07	yes
Breast tumor 299	GSE45725	GPL6883	GSM1113038	4849554043_E	FNA	8.1	51	0.5	IMC	2.2	2	0(2)	(-)	(-)	(+)	(+)	(+)	1	0	38.07	yes
Breast tumor 300	GSE45725	GPL6883	GSM1113039	4849554043_F	FNA	7.2	64	0.7	IDC	2.4	3	0(3)	(-)	(-)	(-)	(-)	(-)	3	0	24.17	yes
Breast tumor 301	GSE45725	GPL6883	GSM1113040	4849554043_G	FNA	8.3	62	0.6	IDC	1.6	2	1(15)	(+)	(-)	(+)	(-)	(-)	1	0	37.77	yes
Breast tumor 302	GSE45725	GPL6883	GSM1113041	4849554043_H	FNA	8	40	0.5	IDC	2.8	3	2(16)	(+)	(-)	(+)	(-)	(-)	2	0	37.57	yes
Breast tumor 303	GSE45725	GPL6883	GSM1113042	4849554044_A	FNA	6.8	59	0.2	IDC	2.1	2	1(28)	(-)	(+)	(-)	(-)	(+)	3	0	17.07	yes
Breast tumor 304	GSE45725	GPL6883	GSM1113043	4849554044_B	FNA	8.5	45	0.033	IDC	2.4	2	0(8)	(-)	(-)	(+)	(+)	(-)	1	0	36.50	yes
Breast tumor 305	GSE45725	GPL6883	GSM1113044	4849554044_C	FNA	8.8	49	0.13	IDC	2.1	2	0(4)	(+)	(-)	(+)	(+)	(-)	2	0	37.63	yes
Breast tumor 306	GSE45725	GPL6883	GSM1113045	4849554044_E	FNA	9.2	44	0.2	IDC	2.6	3	2(4)	(-)	(-)	(-)	(-)	(+)	3	0	40.03	yes
Breast tumor 307	GSE45725	GPL6883	GSM1113046	4849554044_F	FNA	7.8	65	0.6	IDC	2.2	2	0(4)	(-)	(+)	(+)	(+)	(-)	2	0	42.10	yes
Breast tumor 308	GSE45725	GPL6883	GSM1113047	4849554044_G	FNA	8.3	87	0.5	IDC	3.2	3	0(0)	(+)	(-)	(+)	(+)	(-)	2	1	16.20	yes
Breast tumor 309	GSE45725	GPL6883	GSM1113048	4849554044_H	FNA	8.2	72	0.5	IDC	2.1	2	0(1)	(-)	(+)	(+)	(+)	(-)	2	0	32.80	yes
Breast tumor 310	GSE45725	GPL6883	GSM1113049	4849554045_A	FNA	6.5	60	0.175	ILC	1.9	3	1(2)	(+)	(-)	(+)	(+)	(-)	2	0	34.07	yes
Breast tumor 311	GSE45725	GPL6883	GSM1113050	4849554045_B	FNA	8.5	76	1.6	IDC	3.2	2	1(14)	(+)	(-)	(+)	(+)	(-)	1	0	35.03	yes
Breast tumor 312	GSE45725	GPL6883	GSM1113051	4849554045_C	FNA	7.6	44	1.5	IDC	1.1	2	1(21)	(+)	(+)	(+)	(+)	(-)	1	0	34.53	yes
Breast tumor 313	GSE45725	GPL6883	GSM1113052	4849554045_D	FNA	8.9	55	0.22	IDC	1.3	3	0(9)	(-)	(+)	(-)	(-)	(+)	3	0	35.93	yes
Breast tumor 314	GSE45725	GPL6883	GSM1113053	4849554045_E	FNA	8.4	44	NA	ADH	NA	NA	NA	NA	NA	NA	NA	NA	1	0	43.17	no
Breast tumor 315	GSE45725	GPL6883	GSM1113054	4849554045_F	FNA	9.4	39	0.7	IDC	3.7	3	1(23)	(+)	(-)	(+)	(-)	(-)	2	0	38.10	yes
Breast tumor 316	GSE45725	GPL6883	GSM1113055	4849554045_G	FNA	8	46	0	IDC	3	3	2(27)	(+)	(+)	(-)	(-)	(+)	3	1	36.07	yes
Breast tumor 317	GSE45725	GPL6883	GSM1113056	4849554045_H	FNA	7.7	44	3.2	IMC	4.7	2	2(17)	(+)	(-)	(+)	(+)	(-)	1	0	25.20	yes
Breast tumor 318	GSE45725	GPL6883	GSM1113057	4807266013_B	FNA	5.9	63	0.5	IDC	1.2	2	0(3)	(-)	(+)	(-)	(-)	(-)	3	0	39.40	yes
Breast tumor 319	GSE45725	GPL6883	GSM1113058	4807266013_C	FNA	5.6	48	0.2	IDC	4.5	3	0(1)	(+)	(-)	(-)	(-)	(-)	3	0	38.53	yes
Breast tumor 320	GSE45725	GPL6883	GSM1113059	4807266013_D	FNA	5.5	56	0.7	IDC	4.4	3	0(5)	(-)	(-)	(-)	(-)	(-)	3	0	36.27	yes
Breast tumor 321	GSE45725	GPL6883	GSM1113060	4807266013_E	FNA	1.5	42	0.15	IDC	3	1	0(6)	(-)	(-)	(+)	(+)	(+)	1	0	32.37	no
Breast tumor 322	GSE45725	GPL6883	GSM1113061	4807266013_F	FNA	5.4	56	0.33	IDC	1.5	3	0(5)	(-)	(-)	(+)	(-)	(+)	2	0	0.40	no
Breast tumor 323	GSE45725	GPL6883	GSM1113062	4921085012_A	FNA	9.7	60	0.3	IDC	1.8	3	0(5)	(+)	(-)	(+)	(+)	(-)	3	1	9.63	yes
Breast tumor 324	GSE45725	GPL6883	GSM1113063	4921085012_B	FNA	6.5	71	1.2	IDC	2.5	2	0(3)	(-)	(-)	(+)	(+)	(-)	1	0	33.67	yes
Breast tumor 325	GSE45725	GPL6883	GSM1113064	4921085012_C	FNA	8.9	53	NA	IDC	1.6	2	0(2)	(-)	(-)	(+)	(+)	(-)	1	0	34.77	yes
Breast tumor 326	GSE45725	GPL6883	GSM1113065	4921085012_D	FNA	9.4	59	2	IDC	6.4	3	11(31)	(+)	(-)	(+)	(+)	(+)	3	0	36.77	yes
Breast tumor 327	GSE45725	GPL6883	GSM1113066	4921085012_E	FNA	9.3	50	0.4	IDC	1.8	3	7(22)	(+)	(-)	(+)	(+)	(-)	1	0	36.00	yes

Sample_title	GEO_series_accession	GEO_platform_accession	GEO_sample_accession	GEO_raw_data/rep	Sample_type/rep	Sample_RIN/rep	Patient_age	Surgical_margin (cm)	Tumor_type	Tumor_size(cm)	Tumor_grade	Positive_LN(total_LN)	LVI	EIC	ER	PR	HER2	CMTC	Recurrence	Recurrence-free_months	Used_in_the_431BC_dataset?
Breast tumor 328	GSE45725	GPL6883	GSM1113067	4921085012_F	FNA	9	51	0.4	IDC	0.84	1	1(7)	(-)	(-)	(+)	(+)	(-)	1	0	36.00	yes
Breast tumor 329	GSE45725	GPL6883	GSM1113068	4921085012_G	FNA	9.4	45	0.3	IDC	1.7	3	0(3)	(+)	(-)	(+)	(-)	(-)	2	0	30.60	yes
Breast tumor 330	GSE45725	GPL6883	GSM1113069	4921085012_H	FNA	8.9	73	0.1	IDC	3.4	3	0(2)	(+)	(+)	(+)	(+)	(-)	2	0	28.23	yes
Breast tumor 331	GSE45725	GPL6883	GSM1113070	4921106008_A	FNA	8.9	52	0.2	IDC	1.5	3	17(29)	(+)	(-)	(-)	(-)	(-)	3	0	34.93	yes
Breast tumor 332	GSE45725	GPL6883	GSM1113071	4921106008_B	FNA	5.7	57	0.5	IDC	2.6	3	0(3)	(+)	(-)	(-)	(-)	(-)	3	0	36.40	yes
Breast tumor 333	GSE45725	GPL6883	GSM1113072	4921106008_C	FNA	7	72	0.3	IDC	3	3	0(0)	(-)	(-)	(+)	(-)	(-)	3	1	4.53	no
Breast tumor 334	GSE45725	GPL6883	GSM1113073	4921106008_D	FNA	6.8	90	NA	NA	1.90	NA	NA	NA	NA	(+)	(+)	(-)	1	0	10.00	no
Breast tumor 335	GSE45725	GPL6883	GSM1113074	4921106008_E	FNA	8.8	57	0.3	IDC	1.3	3	0(5)	(-)	(-)	(+)	(+)	(-)	2	0	35.30	yes
Breast tumor 336	GSE45725	GPL6883	GSM1113075	4921106008_F	FNA	8.8	50	0.05	IDC	3.9	3	16(19)	(+)	(+)	(-)	(-)	(+)	3	0	35.30	yes
Breast tumor 337	GSE45725	GPL6883	GSM1113076	4921106008_G	FNA	6.2	72	0.5	IDC	2.5	3	0(4)	(-)	(-)	(+)	(+)	(-)	3	0	23.27	yes
Breast tumor 338	GSE45725	GPL6883	GSM1113077	4921106019_A	FNA	6.4	54	0.6	IDC	4.1	2	0(3)	(-)	(-)	(+)	(+)	(-)	1	0	36.13	yes
Breast tumor 339	GSE45725	GPL6883	GSM1113078	4921106019_B	FNA	7.1	55	NA	IDC	3.2	1	3(9)	(+)	(-)	(+)	(+)	(-)	1	0	6.50	yes
Breast tumor 340	GSE45725	GPL6883	GSM1113079	4921106019_C	FNA	7	60	0	DCIS	2.6	NA	0(9)	(-)	(-)	NA	NA	NA	3	0	27.77	no
Breast tumor 341	GSE45725	GPL6883	GSM1113080	4921106019_D	FNA	9.1	70	0.2	DCIS	1.3	NA	0(0)	(-)	(-)	NA	NA	NA	1	0	28.93	no
Breast tumor 342	GSE45725	GPL6883	GSM1113081	4921106019_E	FNA	7.7	54	0.6	IDC	2.4	2	6(24)	(-)	(+)	(+)	(+)	(-)	2	0	35.47	yes
Breast tumor 343	GSE45725	GPL6883	GSM1113082	4921106019_F	FNA	7.4	59	0.1	IDC	1.6	3	0(2)	(-)	(-)	(-)	(-)	(+)	3	0	34.40	yes
Breast tumor 344	GSE45725	GPL6883	GSM1113083	4921106019_G	FNA	8.3	66	0.5	IDC	2.5	3	14(24)	(+)	(-)	(+)	(+)	(+)	2	1	33.70	yes
Breast tumor 345	GSE45725	GPL6883	GSM1113084	4921106019_H	FNA	7.3	52	0	ILC	2.8	2	0(3)	(-)	(-)	(+)	(+)	(-)	1	0	24.57	yes
Breast tumor 346	GSE45725	GPL6883	GSM1113085	4921106020_A	FNA	7.9	85	0.9	IDC	1.2	2	0(3)	(-)	(-)	(+)	(+)	(-)	1	0	2.00	no
Breast tumor 347	GSE45725	GPL6883	GSM1113086	4921106020_B	FNA	8.8	61	0	IDC	0.4	1	0(0)	(-)	(-)	(+)	(+)	(-)	3	0	31.40	yes
Breast tumor 348	GSE45725	GPL6883	GSM1113087	4921106020_D	FNA	8.2	72	0.4	IDC	2.2	3	0(3)	(-)	(-)	(-)	(-)	(+)	3	0	34.83	yes
Breast tumor 349	GSE45725	GPL6883	GSM1113088	4921106020_E	FNA	7.3	53	0.1	IDC	4.5	3	2(17)	(+)	(+)	(+)	(+)	(+)	2	0	35.77	yes
Breast tumor 350	GSE45725	GPL6883	GSM1113089	4921106020_F	FNA	7.1	48	0.3	ILC	3.6	2	2(16)	(-)	(-)	(+)	(+)	(-)	1	0	18.03	yes
Breast tumor 351	GSE45725	GPL6883	GSM1113090	4921106020_G	FNA	8.7	45	0.6	DCIS	0.8	2	0(0)	(-)	(-)	NA	NA	NA	1	0	27.83	no
Breast tumor 352	GSE45725	GPL6883	GSM1113091	4921106020_H	FNA	7.8	73	1	IDC	0.9	3	0(2)	(-)	(-)	(+)	(-)	(-)	2	0	34.63	yes
Breast tumor 353	GSE45725	GPL6883	GSM1113092	4921106025_A	FNA	7.4	55	0.2	IDC	1.1	2	0(5)	(+)	(-)	(+)	(-)	(-)	2	0	35.27	yes
Breast tumor 354	GSE45725	GPL6883	GSM1113093	4921106025_B	FNA	7.2	46	1	DCIS/IDC	0.06	NA	0(4)	(-)	(-)	NA	NA	NA	1	0	20.10	no
Breast tumor 355	GSE45725	GPL6883	GSM1113094	4921106025_C	FNA	8	79	1.3	IDC	1.9	3	0(6)	(-)	(-)	(+)	(+)	(-)	2	0	25.83	yes
Breast tumor 356	GSE45725	GPL6883	GSM1113095	4921106025_E	FNA	6.3	47	0.1	IDC	3.1	3	4(22)	(+)	(-)	(+)	(+)	(-)	2	0	33.67	yes
Breast tumor 357	GSE45725	GPL6883	GSM1113096	4921106025_F	FNA	7.4	43	0.1	IDC	10.5	2	6(21)	(+)	(-)	(+)	(+)	(-)	2	0	32.00	yes
Breast tumor 358	GSE45725	GPL6883	GSM1113097	4921106025_H	FNA	5.3	69	0.2	IDC	1.4	2	0(1)	(-)	(-)	(-)	(-)	(+)	3	0	25.00	yes
Breast tumor 359	GSE45725	GPL6883	GSM1113098	4921106027_A	FNA	8.4	72	0.7	IDC	1.5	2	0(1)	(-)	(-)	(+)	(+)	(-)	1	0	19.10	yes
Breast tumor 360	GSE45725	GPL6883	GSM1113099	4921106027_B	FNA	7.6	45	0.8	IDC	2.7	3	1(33)	(+)	(-)	(+)	(+)	(-)	2	0	34.80	yes
Breast tumor 361	GSE45725	GPL6883	GSM1113100	4921106027_C	FNA	8.8	53	0.5	IDC	1.8	3	0(3)	(-)	(-)	(-)	(-)	(-)	3	0	32.73	yes
Breast tumor 362	GSE45725	GPL6883	GSM1113101	4921106027_D	FNA	7.4	65	NA	IDC	1.9	3	1(18)	(+)	(-)	(+)	(+)	(-)	2	0	34.27	yes
Breast tumor 363	GSE45725	GPL6883	GSM1113102	5083888043_A	FNA	7.8	65	0.1	IDC	2.3	2	2(11)	(+)	(-)	(+)	(+)	(-)	2	0	29.17	yes
Breast tumor 364	GSE45725	GPL6883	GSM1113103	5083888043_B	FNA	8.6	62	0.7	ILC	1.2	2	0(0)	(-)	(-)	(+)	(-)	(-)	1	0	32.23	yes
Breast tumor 365	GSE45725	GPL6883	GSM1113104	5083888043_C	FNA	NA	82	1	IDC	1.6	2	0(11)	(-)	(-)	(-)	(-)	(-)	3	0	30.80	no
Breast tumor 366	GSE45725	GPL6883	GSM1113105	5083888043_D	FNA	7.7	50	0.04	DCIS	2.2	NA	NA	(-)	NA	NA	NA	NA	1	0	30.50	no
Breast tumor 367	GSE45725	GPL6883	GSM1113106	5083888043_E	FNA	7.1	49	0.2	IDC	3	3	10(17)	(+)	(-)	(-)	(-)	(-)	3	1	22.77	yes
Breast tumor 368	GSE45725	GPL6883	GSM1113107	5083888043_F	FNA	7	65	NA	NA	1.80	NA	NA	NA	NA	(+)	(+)	(-)	2	NA	NA	no
Breast tumor 369	GSE45725	GPL6883	GSM1113108	5083888043_G	FNA	6.4	62	2.2	IDC	2.2	3	0(4)	(+)	(-)	(+)	(+)	(-)	1	0	26.20	yes
Breast tumor 370	GSE45725	GPL6883	GSM1113109	5083888043_H	FNA	6.8	61	0.5	DCIS	5.2	NA	0(4)	(+)	(+)	(+)	(-)	(+)	3	0	26.63	no
Breast tumor 371	GSE45725	GPL6883	GSM1113110	5083888049_A	FNA	9	84	0.1	IDC	2.4	3	0(3)	(-)	(-)	(+)	(-)	(+)	2	0	10.50	yes
Breast tumor 372	GSE45725	GPL6883	GSM1113111	5083888049_B	FNA	7.6	55	0.03	IDC	1.5	3	0(5)	(-)	(-)	(+)	(-)	(-)	3	0	18.60	yes
Breast tumor 373	GSE45725	GPL6883	GSM1113112	5083888049_C	FNA	7.5	38	0.2	IDC	2	2	0(2)	(+)	(-)	(+)	(+)	(-)	2	0	33.63	yes
Breast tumor 374	GSE45725	GPL6883	GSM1113113	5083888049_D	FNA	5.9	51	0.8	IMC	3	3	14(44)	(+)	(-)	(-)	(-)	(+)	3	0	25.43	yes

Sample_title	GEO_series_accession	GEO_platform_accession	GEO_sample_accession	GEO_raw_data/rep	Sample_type/rep	Sample_RIN/rep	Patient_age	Surgical_margin (cm)	Tumor_type	Tumor_size(cm)	Tumor_grade	Positive_LN(total_LN)	LVI	EIC	ER	PR	HER2	CMTC	Recurrence	Recurrence-free_months	Used_in_the_431BC_dataset?
Breast tumor 375	GSE45725	GPL6883	GSM1113114	5083888049_E	FNA	7.1	85	0.7	IDC	1.5	2	1(6)	(+)	(-)	(+)	(+)	(-)	1	0	23.97	yes
Breast tumor 376	GSE45725	GPL6883	GSM1113115	5083888049_F	FNA	7.8	62	0.2	IDC	1.8	3	3(15)	(+)	(-)	(+)	(-)	(+)	3	0	32.27	yes
Breast tumor 377	GSE45725	GPL6883	GSM1113116	5083888049_H	FNA	7.6	68	0.3	IDC	1	1	0(2)	(+)	(-)	(+)	(+)	(-)	1	0	31.93	yes
Breast tumor 378	GSE45725	GPL6883	GSM1113117	5083888051_A	FNA	7.2	49	0	IDC	1.2	2	0(3)	(+)	(-)	(+)	(+)	(-)	1	0	27.80	yes
Breast tumor 379	GSE45725	GPL6883	GSM1113118	5083888051_B	FNA	7.5	56	0.6	IDC	0.3	2	0(0)	(-)	(+)	(-)	(-)	(+)	3	0	31.97	yes
Breast tumor 380	GSE45725	GPL6883	GSM1113119	5083888051_C	FNA	7.8	56	0.6	IDC	1.6	3	0(3)	(-)	(+)	(-)	(-)	(+)	3	0	28.97	yes
Breast tumor 381	GSE45725	GPL6883	GSM1113120	5083888051_D	FNA	8.8	48	0.1	IDC	3	3	0(3)	(-)	(-)	(+)	(-)	(+)	2	0	31.97	yes
Breast tumor 382	GSE45725	GPL6883	GSM1113121	5083888051_E	FNA	6.3	61	NA	IDC	0.3	3	0(0)	(-)	(+)	(+)	(-)	(-)	2	0	27.47	yes
Breast tumor 383	GSE45725	GPL6883	GSM1113122	5083888051_F	FNA	7.4	69	NA	IDC	1	2	1(4)	(-)	(-)	(+)	(+)	(-)	1	0	33.53	yes
Breast tumor 384	GSE45725	GPL6883	GSM1113123	5083888051_G	FNA	7.6	72	0.01	IDC	2.1	1	1(16)	(-)	(-)	(+)	(+)	(-)	1	0	29.97	yes
Breast tumor 385	GSE45725	GPL6883	GSM1113124	5083888051_H	FNA	8.3	61	0.1	IDC	0.4	1	0(0)	(-)	(+)	(+)	(+)	(-)	2	0	5.03	no
Breast tumor 386	GSE45725	GPL6883	GSM1113125	5083888055_A	FNA	5.7	60	0	ILC	5.1	3	2(19)	(+)	(-)	(+)	(+)	(-)	2	0	32.90	yes
Breast tumor 387	GSE45725	GPL6883	GSM1113126	5083888055_B	FNA	7.7	80	0.3	DCIS/IDC	1.6	3	NA	NA	NA	(+)	(+)	(+)	1	0	32.13	no
Breast tumor 388	GSE45725	GPL6883	GSM1113127	5083888055_C	FNA	6.2	57	0.2	IDC	1.8	2	0(3)	(-)	(+)	(+)	(-)	(-)	1	0	28.27	yes
Breast tumor 389	GSE45725	GPL6883	GSM1113128	5083888055_D	FNA	7.8	NA	NA	NA	NA	NA	NA	NA	NA	NA	NA	NA	3	0	32.17	no
Breast tumor 390	GSE45725	GPL6883	GSM1113129	5083888055_E	FNA	7.8	32	0.9	IDC	2.5	3	0(7)	(-)	(-)	(-)	(-)	(-)	3	0	32.10	yes
Breast tumor 391	GSE45725	GPL6883	GSM1113130	5083888055_F	FNA	7.6	59	0.2	IDC	3.7	3	20(24)	(+)	(-)	(-)	(-)	(-)	3	0	30.57	yes
Breast tumor 392	GSE45725	GPL6883	GSM1113131	5083888055_G	FNA	6.5	81	0.4	IDC	1.8	2	0(5)	(-)	(-)	(+)	(+)	(-)	3	0	32.87	yes
Breast tumor 393	GSE45725	GPL6883	GSM1113132	5083888055_H	FNA	5.9	50	0.2	IDC	2.8	3	3(18)	(-)	(-)	(-)	(-)	(-)	3	0	31.00	yes
Breast tumor 394	GSE45725	GPL6883	GSM1113133	5083888056_A	FNA	9	48	0.3	IDC	1.6	3	0(3)	(-)	(-)	(+)	(+)	(-)	2	0	26.93	yes
Breast tumor 395	GSE45725	GPL6883	GSM1113134	5083888056_B	FNA	8.8	54	1	ILC	0.9	2	0(3)	(-)	(-)	(-)	(-)	(-)	1	0	25.63	yes
Breast tumor 396	GSE45725	GPL6883	GSM1113135	5083888056_C	FNA	7.9	77	0.3	IDC	3.3	2	0(1)	(-)	(-)	(+)	(-)	(-)	2	0	18.93	yes
Breast tumor 397	GSE45725	GPL6883	GSM1113136	5083888056_D	FNA	8.6	40	0.3	IDC	2.4	3	0(4)	(-)	(+)	(+)	(-)	(+)	3	0	32.00	yes
Breast tumor 398	GSE45725	GPL6883	GSM1113137	5083888056_E	FNA	6.8	61	0.5	IDC	3.1	3	0(4)	(-)	(-)	(-)	(-)	(+)	3	0	30.40	yes
Breast tumor 399	GSE45725	GPL6883	GSM1113138	5083888056_F	FNA	7.7	50	0.5	DCIS	3.7	NA	0(0)	(-)	(-)	NA	NA	NA	3	0	21.07	no
Breast tumor 400	GSE45725	GPL6883	GSM1113139	5083888056_G	FNA	6.3	46	0.4	IDC	2	3	2(4)	(-)	(-)	(-)	(-)	(-)	3	0	29.90	yes
Breast tumor 401	GSE45725	GPL6883	GSM1113140	5083888056_H	FNA	9.3	46	0.1	IDC	1.9	3	1(17)	(-)	(-)	(+)	(-)	(+)	3	0	28.63	yes
Breast tumor 402	GSE45725	GPL6883	GSM1113141	5083888057_A	FNA	9	82	<0.05	IDC/DCIS	1.9	3	0(3)	(-)	(-)	(+)	(-)	(-)	3	0	26.63	yes
Breast tumor 403	GSE45725	GPL6883	GSM1113142	5083888057_B	FNA	7.1	41	0.1	IDC	2	3	2(19)	(+)	(-)	(+)	(+)	(-)	2	0	32.33	yes
Breast tumor 404	GSE45725	GPL6883	GSM1113143	5083888057_C	FNA	6.1	61	0.5	IDC	2.5	2	0(2)	(+)	(-)	(-)	(-)	(+)	3	0	28.43	yes
Breast tumor 405	GSE45725	GPL6883	GSM1113144	5083888057_D	FNA	6.5	70	1	ILC	1.9	2	1(11)	(-)	(-)	(+)	(+)	(-)	1	0	23.90	yes
Breast tumor 406	GSE45725	GPL6883	GSM1113145	5083888057_E	FNA	8.2	54	0.2	IDC	2.6	3	3(13)	(+)	(-)	(+)	(-)	(-)	2	0	30.63	yes
Breast tumor 407	GSE45725	GPL6883	GSM1113146	5083888057_F	FNA	9.6	59	1	IDC	3.7	2	6(14)	(+)	(-)	(+)	(+)	(+)	2	0	23.37	yes
Breast tumor 408	GSE45725	GPL6883	GSM1113147	5083888057_G	FNA	7.1	49	NA	NA	NA	NA	NA	NA	NA	NA	NA	NA	2	0	31.97	no
Breast tumor 409	GSE45725	GPL6883	GSM1113148	5083888057_H	FNA	7.9	61	0.2	IDC	1.4	1	0(5)	(+)	(-)	(+)	(+)	(-)	1	0	32.13	yes
Breast tumor 410	GSE45725	GPL6883	GSM1113149	4936527003_A	FNA	5.1	66	0.4	IDC	3.6	3	20(28)	(+)	(+)	(+)	(+)	(-)	2	0	31.93	yes
Breast tumor 411	GSE45725	GPL6883	GSM1113150	4936527003_B	FNA	7.4	49	0.3	IDC	1.8	1	0(1)	(-)	(-)	(+)	(+)	(-)	1	0	28.30	yes
Breast tumor 412	GSE45725	GPL6883	GSM1113151	4936527003_C	FNA	9.4	64	0.6	IDC	2.7	2	1(13)	(+)	(-)	(-)	(-)	(+)	3	0	29.83	yes
Breast tumor 413	GSE45725	GPL6883	GSM1113152	4936527003_D	FNA	9.2	61	0.4	IDC	1.5	2	0(1)	(-)	(-)	(+)	(-)	(-)	2	0	31.30	yes
Breast tumor 414	GSE45725	GPL6883	GSM1113153	4936527003_E	FNA	7.8	53	0.3	IDC	3	3	21(38)	(-)	(-)	(+)	(+)	(-)	1	0	11.60	yes
Breast tumor 415	GSE45725	GPL6883	GSM1113154	4936527003_F	FNA	7.6	73	0.5	IDC	2.2	2	1(12)	(+)	(-)	(+)	(+)	(-)	1	0	28.80	yes
Breast tumor 416	GSE45725	GPL6883	GSM1113155	4936527003_G	FNA	NA	44	1	IDC	2.8	2	0(5)	(+)	(-)	(+)	(-)	(-)	2	0	12.03	no
Breast tumor 417	GSE45725	GPL6883	GSM1113156	4936527026_A	FNA	9.6	81	0.9	IDC	1.5	1	0(4)	(+)	(-)	(+)	(+)	(-)	2	0	31.40	yes
Breast tumor 418	GSE45725	GPL6883	GSM1113157	4936527026_B	FNA	8.2	70	0.9	IDC	2.6	1	4(21)	(-)	(-)	(+)	(+)	(-)	1	0	30.87	yes
Breast tumor 419	GSE45725	GPL6883	GSM1113158	4936527026_C	FNA	9.6	63	0.2	IDC	1.3	2	2(47)	(-)	(-)	(-)	(-)	(-)	3	0	29.17	yes
Breast tumor 420	GSE45725	GPL6883	GSM1113159	4936527026_E	FNA	7.7	58	0.6	IDC	0.7	2	0(6)	(+)	(-)	(+)	(-)	(-)	3	0	24.43	yes
Breast tumor 421	GSE45725	GPL6883	GSM1113160	4936527026_F	FNA	6.8	44	0.5	IMC	0.9	1	0(3)	(-)	(-)	(+)	(+)	(-)	1	0	9.47	yes

Sample_title	GEO_series_accession	GEO_platform_accession	GEO_sample_accession	GEO_raw_data/rep	Sample_type/rep	Sample_RIN/rep	Patient_age	Surgical_margin (cm)	Tumor_type	Tumor_size(cm)	Tumor_grade	Positive_LN(total_LN)	LVI	EIC	ER	PR	HER2	CMTC	Recurrence	Recurrence-free_months	Used_in_the_431BC_dataset?
Breast tumor 422	GSE45725	GPL6883	GSM1113161	4936527026_G	FNA	7	40	NA	DCIS	1.5	NA	NA	NA	NA	NA	NA	NA	1	0	28.30	no
Breast tumor 423	GSE45725	GPL6883	GSM1113162	4936527026_H	FNA	8.1	61	0.5	IDC	0.4	2	0(0)	(-)	(-)	(+)	(+)	(-)	2	0	21.40	yes
Breast tumor 424	GSE45725	GPL6883	GSM1113163	5392306038_A	FNA	7.2	45	0.5	IDC	0.2	2	3(7)	(+)	(+)	(+)	(+)	(-)	1	0	27.80	yes
Breast tumor 425	GSE45725	GPL6883	GSM1113164	5392306038_B	FNA	6.6	49	0.6	IDC	2	3	2(23)	(-)	(-)	(-)	(-)	(+)	3	0	21.50	yes
Breast tumor 426	GSE45725	GPL6883	GSM1113165	5392306038_C	FNA	9.1	48	0.3	IDC	3.2	2	1(12)	(-)	(-)	(+)	(+)	(-)	2	0	30.33	yes
Breast tumor 427	GSE45725	GPL6883	GSM1113166	5392306038_D	FNA	7.4	94	0.1	IDC	2.1	3	0(3)	(-)	(-)	(+)	(+)	(+)	2	0	25.83	yes
Breast tumor 428	GSE45725	GPL6883	GSM1113167	5392306038_E	FNA	6.9	51	0.5	IDC	3.5	2	0(3)	(-)	(-)	(+)	(+)	(-)	1	0	28.90	yes
Breast tumor 429	GSE45725	GPL6883	GSM1113168	5392306038_F	FNA	8.3	74	0.2	IDC	2.2	1	0(4)	(-)	(-)	(+)	(+)	(-)	1	0	28.43	yes
Breast tumor 430	GSE45725	GPL6883	GSM1113169	5392306038_G	FNA	8.8	53	0.3	IDC	2.1	3	0(3)	(-)	(-)	(+)	(+)	(-)	2	0	25.00	yes
Breast tumor 431	GSE45725	GPL6883	GSM1113170	5392306038_H	FNA	7.8	62	NA	IDC	1.5	2	0(3)	(-)	(-)	(+)	(+)	(-)	1	0	30.87	yes
Breast tumor 432	GSE45725	GPL6883	GSM1113171	5392306045_A	FNA	8.9	57	0.3	IDC	3.2	2	0(22)	(-)	(-)	(+)	(+)	(-)	1	0	28.87	yes
Breast tumor 433	GSE45725	GPL6883	GSM1113172	5392306045_B	FNA	7.7	73	NA	ILC	2.2	2	0(5)	(+)	(-)	(+)	(+)	(-)	2	0	29.13	yes
Breast tumor 434	GSE45725	GPL6883	GSM1113173	5392306045_C	FNA	7.6	53	NA	IDC	0.1	NA	0(4)	(-)	(-)	(-)	(-)	(+)	3	0	3.87	no
Breast tumor 435	GSE45725	GPL6883	GSM1113174	5392306045_D	FNA	9.6	50	0.09	IDC	1.1	3	0(16)	(+)	(-)	(+)	(+)	(-)	2	0	27.00	yes
Breast tumor 436	GSE45725	GPL6883	GSM1113175	5392306045_E	FNA	8.7	64	0.7	IDC	1.8	3	0(5)	(-)	(-)	(+)	(+)	(-)	3	0	23.83	yes
Breast tumor 437	GSE45725	GPL6883	GSM1113176	5392306045_F	FNA	7.5	48	0.01	DCIS	1.6	NA	NA	NA	NA	NA	NA	NA	1	0	27.60	no
Breast tumor 438	GSE45725	GPL6883	GSM1113177	5392306045_G	FNA	8.5	53	0.13	IDC	3.8	2	6(32)	(+)	(-)	(+)	(+)	(-)	2	NA	NA	no
Breast tumor 439	GSE45725	GPL6883	GSM1113178	5392306045_H	FNA	8	75	0.3	IDC	3	3	0(7)	(-)	(-)	(+)	(+)	(-)	2	1	23.87	yes
Breast tumor 440	GSE45725	GPL6883	GSM1113179	5392306052_A	FNA	7.8	49	0.1	IDC	1.2	1	0(0)	(-)	(+)	(+)	(+)	(-)	2	0	26.90	yes
Breast tumor 441	GSE45725	GPL6883	GSM1113180	5392306052_B	FNA	5.7	42	0.225	IDC/DCIS	2.6	3	0(4)	(-)	(-)	(-)	(-)	(-)	3	0	26.33	yes
Breast tumor 442	GSE45725	GPL6883	GSM1113181	5392306052_C	FNA	6.9	56	0.5	IDC	4.8	3	0(9)	(-)	(-)	(+)	(+)	(-)	2	0	24.43	yes
Breast tumor 443	GSE45725	GPL6883	GSM1113182	5392306052_D	FNA	6.8	58	>0.7	DCIS/IDC	5.8	3	1(2)	(+)	(+)	(-)	(-)	(-)	3	0	13.60	no
Breast tumor 444	GSE45725	GPL6883	GSM1113183	5392306052_E	FNA	7.2	48	0.75	IDC	2.2	2	0/3	(+)	(+)	(+)	(+)	(-)	2	0	27.13	yes
Breast tumor 445	GSE45725	GPL6883	GSM1113184	5392306052_F	FNA	9.1	58	0.1	ILC	10	2	0(10)	(-)	(-)	(+)	(-)	(-)	2	0	24.63	yes
Breast tumor 446	GSE45725	GPL6883	GSM1113185	5392306052_G	FNA	9	80	0	ILC	5.7	3	1(11)	(-)	(-)	(+)	(+)	(-)	2	0	26.03	yes
Breast tumor 447	GSE45725	GPL6883	GSM1113186	5392306052_H	FNA	8.4	45	0.5	IDC	1.5	2	0(2)	(-)	(-)	(+)	(+)	(-)	1	0	25.00	yes
Breast tumor 448	GSE45725	GPL6883	GSM1113187	5392306053_A	FNA	9.4	54	0.5	IMC	1.8	2	0(5)	(-)	(-)	(+)	(+)	(-)	1	0	19.40	yes
Breast tumor 449	GSE45725	GPL6883	GSM1113188	5392306053_B	FNA	9.2	62	2	IDC	1.1	2	0(4)	(-)	(-)	(+)	(+)	(-)	2	0	25.17	yes
Breast tumor 450	GSE45725	GPL6883	GSM1113189	5392306053_C	FNA	8.6	44	0.08	ILC	1.4	2	1(25)	(-)	(-)	(+)	(+)	(-)	1	0	22.63	yes
Breast tumor 451	GSE45725	GPL6883	GSM1113190	5392306053_D	FNA	5.7	67	0.4	IDC	1.6	3	1(3)	(+)	(-)	(+)	(-)	(+)	2	0	27.33	yes
Breast tumor 452	GSE45725	GPL6883	GSM1113191	5392306053_E	FNA	7.3	38	0.2	DCIS/IDC	0.1	NA	0(2)	(-)	(-)	(+)	(-)	(-)	3	0	28.30	no
Breast tumor 453	GSE45725	GPL6883	GSM1113192	5392306053_F	FNA	7.4	55	0.2	IDC	1.5	3	0(1)	(-)	(-)	(+)	(-)	(-)	2	0	26.70	yes
Breast tumor 454	GSE45725	GPL6883	GSM1113193	5392306053_G	FNA	8.2	51	0.3	IDC	1.6	1	1(20)	(-)	(-)	(+)	(+)	(-)	1	0	27.80	yes
Breast tumor 455	GSE45725	GPL6883	GSM1113194	5392306053_H	FNA	6.7	55	0.1	IDC	1.7	2	0(3)	(-)	(-)	(+)	(+)	(-)	2	0	3.70	no
Breast tumor 456	GSE45725	GPL6883	GSM1113195	5371062047_A	FNA	7.1	53	0.7	IDC	0.8	3	0(2)	(-)	(-)	(+)	(+)	(+)	3	0	13.03	yes
Breast tumor 457	GSE45725	GPL6883	GSM1113196	5371062047_C	FNA	8.9	50	0.2	IDC	1.8	3	0(3)	(-)	(+)	(+)	(+)	(-)	3	0	21.63	yes
Breast tumor 458	GSE45725	GPL6883	GSM1113197	5371062047_D	FNA	6.9	39	0.3	IDC	1.3	3	0/3	(-)	(-)	(+)	(+)	(-)	1	0	18.33	yes
Breast tumor 459	GSE45725	GPL6883	GSM1113198	5371062047_E	FNA	6.6	42	0.1	IDC	0.9	1	0(2)	(-)	(-)	(+)	(+)	(-)	1	0	5.23	no
Breast tumor 460	GSE45725	GPL6883	GSM1113199	5371062047_F	FNA	8.6	59	NA	DCIS	3.3	NA	0(0)	NA	NA	NA	NA	NA	2	0	27.87	no
Breast tumor 461	GSE45725	GPL6883	GSM1113200	5371062047_G	FNA	6.8	61	0.8	IDC	2.2	3	0(10)	(-)	(-)	(+)	(-)	(+)	2	0	26.57	yes
Breast tumor 462	GSE45725	GPL6883	GSM1113201	5371062047_H	FNA	8	71	0.02	IDC	2.2	3	0(4)	(+)	(-)	(+)	(+)	(-)	2	0	26.90	yes
Breast tumor 463	GSE45725	GPL6883	GSM1113202	5371062058_A	FNA	NA	65	0.08	IDC	4.8	3	0(2)	(+)	(-)	(-)	(-)	(-)	3	1	12.30	no
Breast tumor 464	GSE45725	GPL6883	GSM1113203	5371062058_B	FNA	6.4	76	0.1	IDC	2.9	3	1(4)	(+)	(-)	(-)	(-)	(-)	3	0	13.57	yes
Breast tumor 465	GSE45725	GPL6883	GSM1113204	5371062058_C	FNA	7.9	50	0.1	IDC	1	2	0(2)	(+)	(-)	(+)	(+)	(-)	2	0	22.67	yes
Breast tumor 466	GSE45725	GPL6883	GSM1113205	5371062058_D	FNA	6.1	62	0.3	IDC	2.8	3	0(6)	(+)	(-)	(-)	(-)	(-)	3	0	25.83	yes
Breast tumor 467	GSE45725	GPL6883	GSM1113206	5371062058_E	FNA	5.9	51	1	IDC	3.8	3	1(7)	(+)	(-)	(+)	(-)	(-)	2	0	26.57	yes
Breast tumor 468	GSE45725	GPL6883	GSM1113207	5371062058_F	FNA	6.8	76	NA	IDC	2.7	1	2(22)	(-)	(-)	(+)	(+)	(-)	1	0	0.50	no

Sample_title	GEO_series_accession	GEO_platform_accession	GEO_sample_accession	GEO_raw_data/rep	Sample_type/rep	Sample_RIN/rep	Patient_age	Surgical_margin (cm)	Tumor_type	Tumor_size(cm)	Tumor_grade	Positive_LN(total_LN)	LVI	EIC	ER	PR	HER2	CMTC	Recurrence	Recurrence-free_months	Used_in_the_431BC_dataset?
Breast tumor 469	GSE45725	GPL6883	GSM1113208	5371062058_G	FNA	6.6	74	0.5	IDC	2	3	0(3)	(-)	(-)	(+)	(+)	(-)	2	0	21.93	yes
Breast tumor 470	GSE45725	GPL6883	GSM1113209	5371062058_H	FNA	8.3	66	NA	IDC	2.3	3	10(26)	(+)	(-)	(+)	(+)	(-)	2	NA	NA	no
Breast tumor 471	GSE45725	GPL6883	GSM1113210	5392306058_A	FNA	8.5	47	0.1	IDC	4.8	3	0(3)	(-)	(-)	(+)	(+)	(-)	2	0	24.53	yes
Breast tumor 472	GSE45725	GPL6883	GSM1113211	5392306058_B	FNA	7.6	49	0.1	IDC	4.8	3	3(21)	(+)	(-)	(-)	(-)	(+)	3	0	26.13	yes
Breast tumor 473	GSE45725	GPL6883	GSM1113212	5392306058_D	FNA	9.2	43	NA	NA	NA	NA	NA	NA	NA	NA	NA	NA	1	0	18.33	no
Breast tumor 474	GSE45725	GPL6883	GSM1113213	5392306058_E	FNA	8.7	55	0.3	IDC	1.4	3	0(2)	(+)	(-)	(+)	(+)	(-)	2	0	24.07	yes
Breast tumor 475	GSE45725	GPL6883	GSM1113214	5392306058_F	FNA	9	71	0.8	IDC	1.1	3	0(2)	(-)	(-)	(+)	(+)	(-)	1	0	25.00	yes
Breast tumor 476	GSE45725	GPL6883	GSM1113215	5392306058_G	FNA	9	59	NA	DCIS	5	NA	0(0)	NA	NA	NA	NA	NA	1	0	23.43	no
Breast tumor 477	GSE45725	GPL6883	GSM1113216	5392306058_H	FNA	6.9	41	0.6	IDC	1.9	3	3(19)	(-)	(-)	(-)	(-)	(-)	3	0	22.70	yes
Breast tumor 478	GSE45725	GPL6883	GSM1113217	5392306060_A	FNA	7.2	74	1.1	IDC	1.2	3	0(24)	(-)	(+)	(+)	(+)	(-)	2	0	26.63	yes
Breast tumor 479	GSE45725	GPL6883	GSM1113218	5392306060_B	FNA	6.1	64	0.3	IDC	1.7	2	0(2)	(-)	(-)	(+)	(-)	(+)	3	0	6.57	yes
Breast tumor 480	GSE45725	GPL6883	GSM1113219	5392306060_C	FNA	8.6	70	0.08	IDC	4	3	0(8)	(-)	(-)	(-)	(-)	(-)	3	NA	NA	no
Breast tumor 481	GSE45725	GPL6883	GSM1113220	5392306060_D	FNA	7.1	70	NA	IDC	1	3	0(2)	(-)	(-)	(-)	(-)	(-)	3	0	25.00	yes
Breast tumor 482	GSE45725	GPL6883	GSM1113221	5392306060_E	FNA	7.8	56	1.2	IDC	3	3	0(6)	(-)	(-)	(+)	(+)	(-)	2	0	23.57	yes
Breast tumor 483	GSE45725	GPL6883	GSM1113222	5392306060_F	FNA	8	71	2	IDC	7	2	4(20)	(+)	(-)	(+)	(+)	(-)	1	0	26.40	yes
Breast tumor 484	GSE45725	GPL6883	GSM1113223	5392306060_G	FNA	6.4	58	0	IDC	1.6	3	2(20)	(-)	(-)	(+)	(-)	(-)	2	0	25.70	yes
Breast tumor 485	GSE45725	GPL6883	GSM1113224	5392306060_H	FNA	8.8	43	0.2	IDC	2.2	2	0(1)	(-)	(-)	(+)	(+)	(-)	1	0	26.13	yes
Breast tumor 486	GSE45725	GPL6883	GSM1113225	5542829005_A	FNA	6.5	47	0.5	IDC	3.7	3	3(20)	(+)	(-)	(+)	(+)	(-)	2	0	25.43	yes
Breast tumor 487	GSE45725	GPL6883	GSM1113226	5542829005_B	FNA	8.2	86	0.3	IDC	2.1	1	0(2)	(-)	(+)	(+)	(+)	(-)	1	0	26.77	yes
Breast tumor 488	GSE45725	GPL6883	GSM1113227	5542829005_C	FNA	6.4	46	1	IDC	3.8	3	1(18)	(+)	(-)	(-)	(-)	(-)	3	0	24.77	yes
Breast tumor 489	GSE45725	GPL6883	GSM1113228	5542829005_D	FNA	9.2	58	0.6	IDC	1.3	2	1(20)	(+)	(-)	(+)	(+)	(-)	1	0	26.10	yes
Breast tumor 490	GSE45725	GPL6883	GSM1113229	5542829005_E	FNA	5.8	87	0.5	IDC	2.1	3	0(2)	(-)	(+)	(-)	(-)	(-)	3	1	9.83	yes
Breast tumor 491	GSE45725	GPL6883	GSM1113230	5542829005_F	FNA	6.7	41	0.4	IDC	4.4	2	2(22)	(+)	(-)	(+)	(-)	(-)	1	0	23.40	yes
Breast tumor 492	GSE45725	GPL6883	GSM1113231	5542829005_G	FNA	8.6	44	0.29	IDC	2.1	2	1(5)	(-)	(-)	(+)	(-)	(-)	2	0	25.90	yes
Breast tumor 493	GSE45725	GPL6883	GSM1113232	5542829005_H	FNA	7	50	1	IDC	1.8	3	0(2)	(+)	(-)	(+)	(+)	(+)	2	0	23.93	yes
Breast tumor 494	GSE45725	GPL6883	GSM1113233	5542829016_A	FNA	7.1	61	1	DCIS	0.03	2	0(0)	NA	NA	NA	NA	NA	2	0	25.53	no
Breast tumor 495	GSE45725	GPL6883	GSM1113234	5542829016_B	FNA	6.2	58	1.2	IDC	2.2	2	1(18)	(+)	(-)	(+)	(+)	(-)	1	0	21.07	yes
Breast tumor 496	GSE45725	GPL6883	GSM1113235	5542829016_C	FNA	8.2	55	0	IDC	0.2	2	0(0)	(-)	(+)	(+)	(+)	(-)	1	0	23.53	yes
Breast tumor 497	GSE45725	GPL6883	GSM1113236	5542829016_D	FNA	7.8	77	0.2	IDC	0.1	2	0(0)	(+)	(+)	(+)	(+)	(-)	2	0	13.33	yes
Breast tumor 498	GSE45725	GPL6883	GSM1113237	5542829016_E	FNA	8.9	59	0.5	IDC	1.6	3	0(3)	(+)	(-)	(+)	(+)	(-)	2	0	23.47	yes
Breast tumor 499	GSE45725	GPL6883	GSM1113238	5542829016_F	FNA	7.7	62	0.3	IDC	2.1	2	0(3)	(-)	(-)	(+)	(+)	(-)	2	0	27.73	yes
Breast tumor 500	GSE45725	GPL6883	GSM1113239	5542829016_G	FNA	7.5	60	0	IDC	5.5	2	2(22)	(+)	(+)	(+)	(+)	(-)	1	0	23.63	yes
Breast tumor 501	GSE45725	GPL6883	GSM1113240	5542829016_H	FNA	7.6	38	0.3	IDC	1.4	2	0(2)	(+)	(-)	(+)	(+)	(-)	2	0	22.70	yes
