# Supplementary material for: Validation of the prognostic gene portfolio, ClinicoMolecular Triad Classification, using an independent prospective breast cancer cohort and external patient populations
Source: Breast Cancer Res. 2014 Jul 4;16(4):R71. doi: 10.1186/bcr3686 (PMC4226941; doi:10.1186/bcr3686)
Supplement: Additional file 2: Table S2 — Comparison of Cox proportional hazards in receptor status, CMTC, subtype and other gene expression signatures as prognostic indicators for recurrence in the 284 internal and 2,181 external breast cancer patients. Figure S1. Pairwise comparisons of CMTC with gene signatures and oncogenic signaling pathway activities between the internal training cohort and validation cohort. Figure S2. Comparison of the prognostic significance between receptor status, molecular subtypes and CMTC in the 4,851 overall breast cancers. Figure S3. The assessment of the prognostic significance of CMTC among different groups based on the receptor status in the 4,851 overall breast cancers. Figure S4. The assessment of the prognostic significance of CMTC among different groups based on other clinicopathologic variables in the 3,963 breast cancers with complete clinical information. Figure S5. The assessment of the prognostic significance of CMTC among different groups based on different clinical variable status in the 4,851 overall breast cancers. Figure S6. The assessment of the prognostic significance of CMTC among different groups based on the different microarray platforms in the 4,851 overall breast cancers. [file bcr3686-S2.pdf]

Supplementary Table S2. Compare of Cox Proportional Hazards in receptor status, CMTC, subtype and other gene expression signatures as prognostic indicators for recurrence in the 284 internal and the 2181 external breast cancer patients

Variables		Internal validation cohort (n=284)			External validation cohort (n=2181)		
		Hazard Ratio	95% CI	*P* value	Hazard Ratio	95% CI	*P* value
Receptor Status	ER	1.89	0.71-5.05	2.00E-01	0.54	0.46-0.64	3.80E-13
	PR	2.12	0.84-5.37	1.10E-01	0.53	0.45-0.63	7.60E-14
	HER2	0.88	0.29-2.66	8.20E-01	1.98	1.63-2.41	9.20E-12
	TN	3.17	1.13-8.91	2.80E-02	1.50	1.23-1.83	5.50E-05
	HER2+/TN	2.24	0.89-5.65	8.70E-02	1.99	1.69-2.34	2.20E-16
CMTC	1 vs. 2	5.66	0.68-47.0	1.10E-01	2.48	1.97-3.12	1.10E-14
	1 vs. 3	12.55	1.62-97.2	1.50E-02	3.28	2.64-4.06	0.00E+00
	1 vs. 2+3	8.79	1.17-66.0	3.50E-02	2.90	2.37-3.55	0.00E+00
PAM50*	1 vs. 2	4.89	0.62-38.6	1.30E-01	2.33	1.89-2.89	5.80E-15
	1 vs. 3	6.31	0.79-50.5	8.20E-02	2.82	2.31-3.44	0.00E+00
	1 vs. 2+3	5.47	0.73-41.1	9.90E-02	2.58	2.15-3.10	0.00E+00
Subtype*	1 vs. 2	2.22	0.56-8.88	2.60E-01	1.97	1.60-2.43	2.20E-10
	1 vs. 3	5.87	1.59-21.7	8.00E-03	2.38	1.97-2.89	0.00E+00
	1 vs. 2+3	3.54	1.03-12.2	4.60E-02	2.19	1.84-2.60	0.00E+00
Gene Signature**	37GS	1.44	0.51-4.03	4.90E-01	1.63	1.36-1.96	1.50E-07
	70GS	5.89	1.94-17.9	1.80E-03	2.00	1.70-2.36	1.10E-16
	76GS	2.11	0.82-5.45	1.20E-01	1.91	1.62-2.24	6.30E-15
	97GS	5.51	1.59-19.0	7.00E-03	2.50	2.11-2.96	0.00E+00
	ERGS	5.98	1.38-26.0	1.70E-02	3.06	2.52-3.73	0.00E+00
	ESGS	4.54	1.31-15.7	1.70E-02	1.69	1.44-1.99	2.10E-10
	IGS	11.30	1.50-84.9	1.80E-02	2.57	2.00-3.31	2.50E-13
	P53GS	3.85	1.12-13.3	3.30E-02	1.94	1.61-2.34	3.20E-12
	Proliferation	2.45	0.95-6.33	6.40E-02	1.93	1.64-2.27	1.90E-15
	SDPP	3.75	1.48-9.51	5.30E-03	2.06	1.75-2.42	0.00E+00
	TGFβRII	0.92	0.36-2.34	8.70E-01	1.37	1.17-1.61	1.40E-04
	WS	1.88	0.67-5.28	2.30E-01	1.96	1.65-2.32	8.90E-15

*Tumor were reset: normal-like and luminal A as group 1, Luminal B as group 2, and basal-like and Her2 as group 3. **Tumors were dichotomized into good and poor prognosis groups based on the correlation coefficient value of zero as the threshold. See Figure 2 for names of the variables and signatures in details.

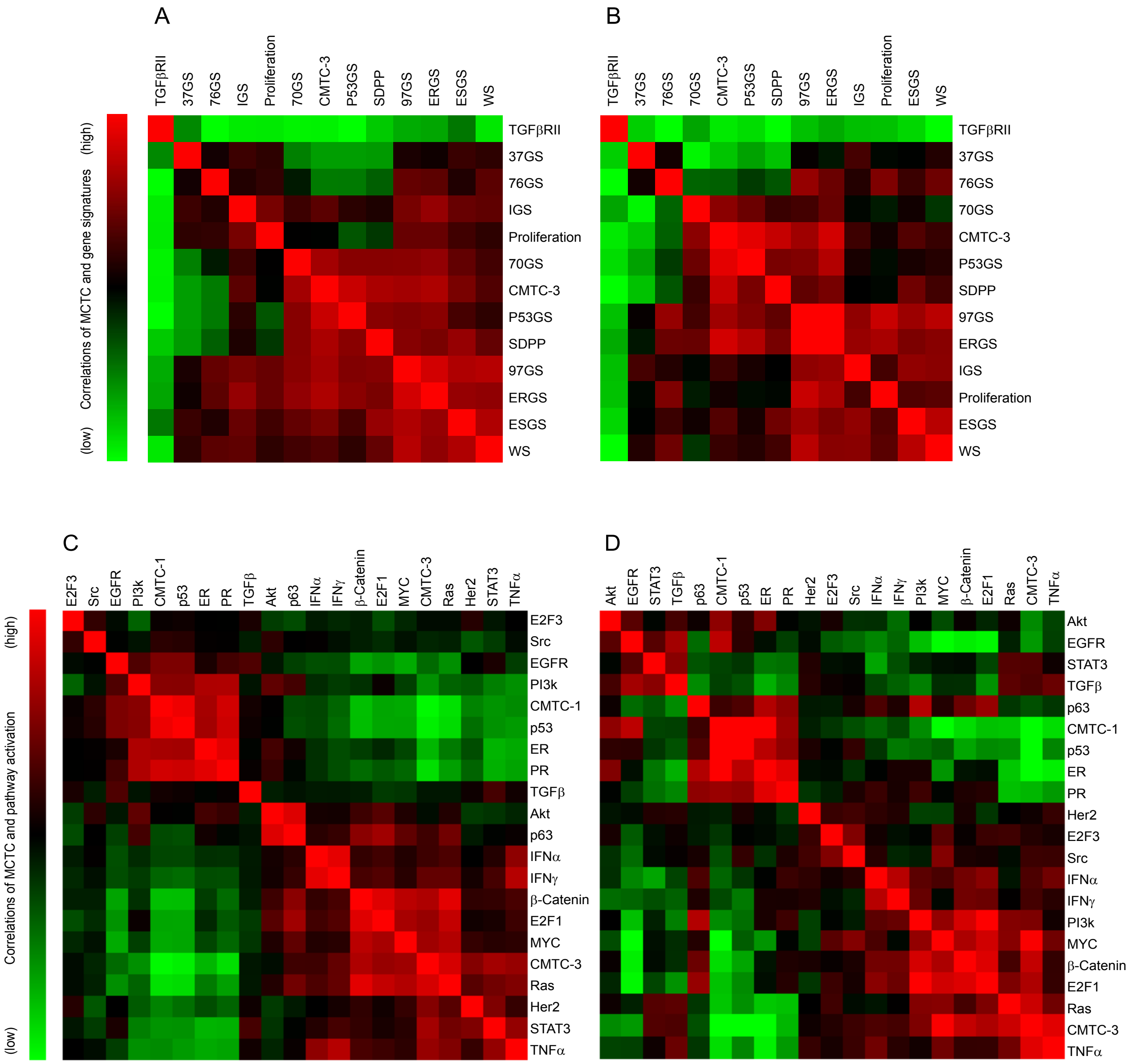

Supplementary Figure S1. Pairwise comparisons of *CMTC* with gene signatures and oncogenic signaling pathway activities between the internal training cohort and validation cohort. Pairwise correlations of CMTC and (**A**) 12 gene signatures and (**C**) 19 oncogenic pathways in the 149 breast cancers in original internal training cohort (Figure 1A in reference [4]); and (**B**) 12 gene signatures and (**D**) 19 oncogenic pathways in the 284 breast cancers in internal validation cohort (Figure 2B). The colors indicate the correlation coefficient values between the centroid values of CMTC3 with the scores of the 12 different gene signatures and 19 different oncogenic pathways: red, positive correlations; and green, negative correlations.

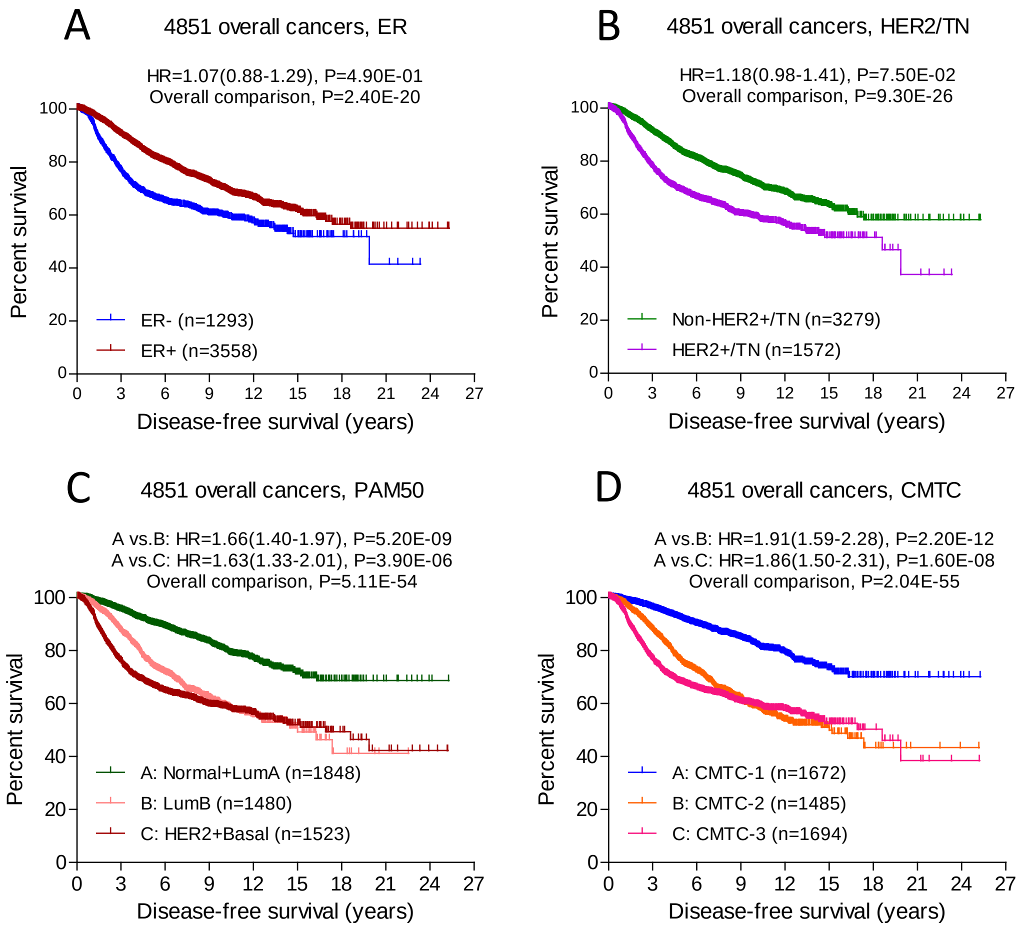

Supplementary Figure S2. Comparison of the prognostic significance between receptor status, molecular subtypes and *CMTC* in the 4851 overall breast cancers. The overall cohort included all internal and external cohorts with a median follow-up of 6.19 years. (**A**) ER status; (**B**) HER2/TN status; (**C**) PAM050- based subtype grouping of the tumors into three groups similar to CMTC: group 1 included normal-like plus luminal A subtypes, group 2 includes luminal B, and group 3 includes HER2 and basal-like subtypes; and **(D)** Three CMTC groups. The HR (hazard ratios) with 95% confidence intervals in parentheses and the *P* values were calculated using the Cox proportional hazards method, multivariate analyses. The *P* values were determined using Log-rank test based on overall comparison.

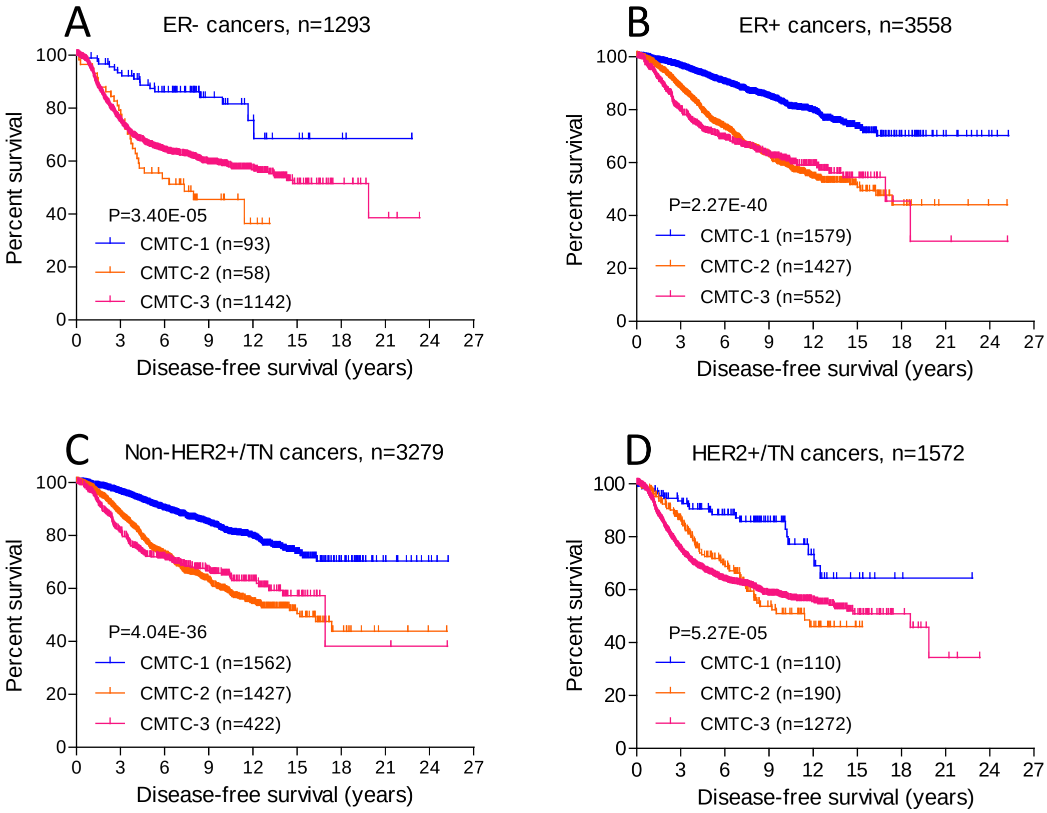

Supplementary Figure S3. The assessment of the prognostic significance of *CMTC* among different groups based on the receptor status in the 4851 overall breast cancers. The overall cohort included patients from all internal and external cohorts. CMTC prognostic predictions among (**A**) ER- cancers; (**B**) ER+ cancers; (**C**) Non-HER2/TN cancers; and (**D**) HER2+/TN cancers. The *P* values were determined using Log-rank test based on overall comparison.

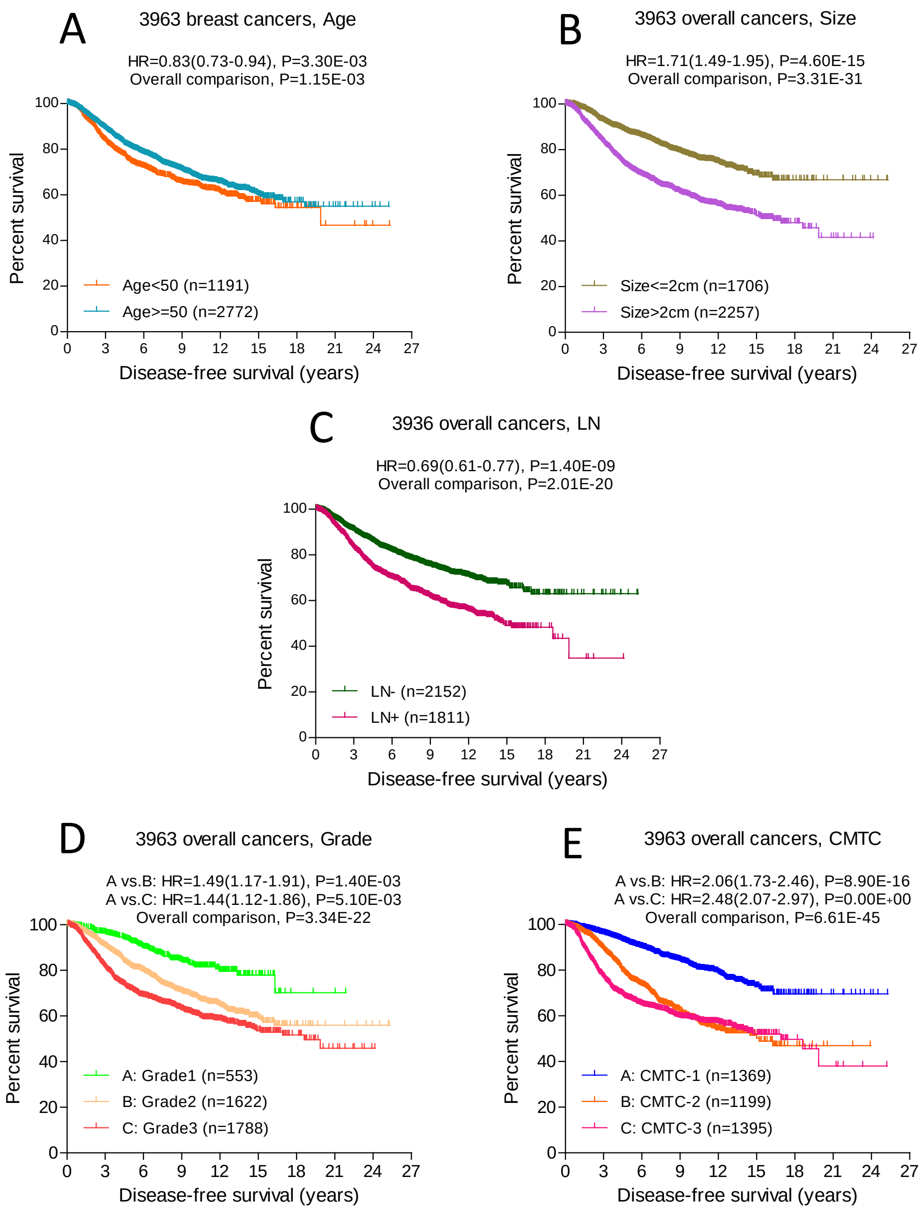

Supplementary Figure S4. The assessment of the prognostic significance of *CMTC* among different groups based on other clinicopathologic variables in the 3963 breast cancers with complete clinical information. (**A**) Patient's age; (**B**) Tumor size; (**C**) Lymph node status; (**D**) Tumor grade status; and **(E)** CMTC. The HR (hazard ratios) with 95% confidence intervals in parentheses and the *P* values were calculated using the Cox proportional hazards method, multivariate analyses. The *P* values were determined using Log-rank test based on overall comparison.

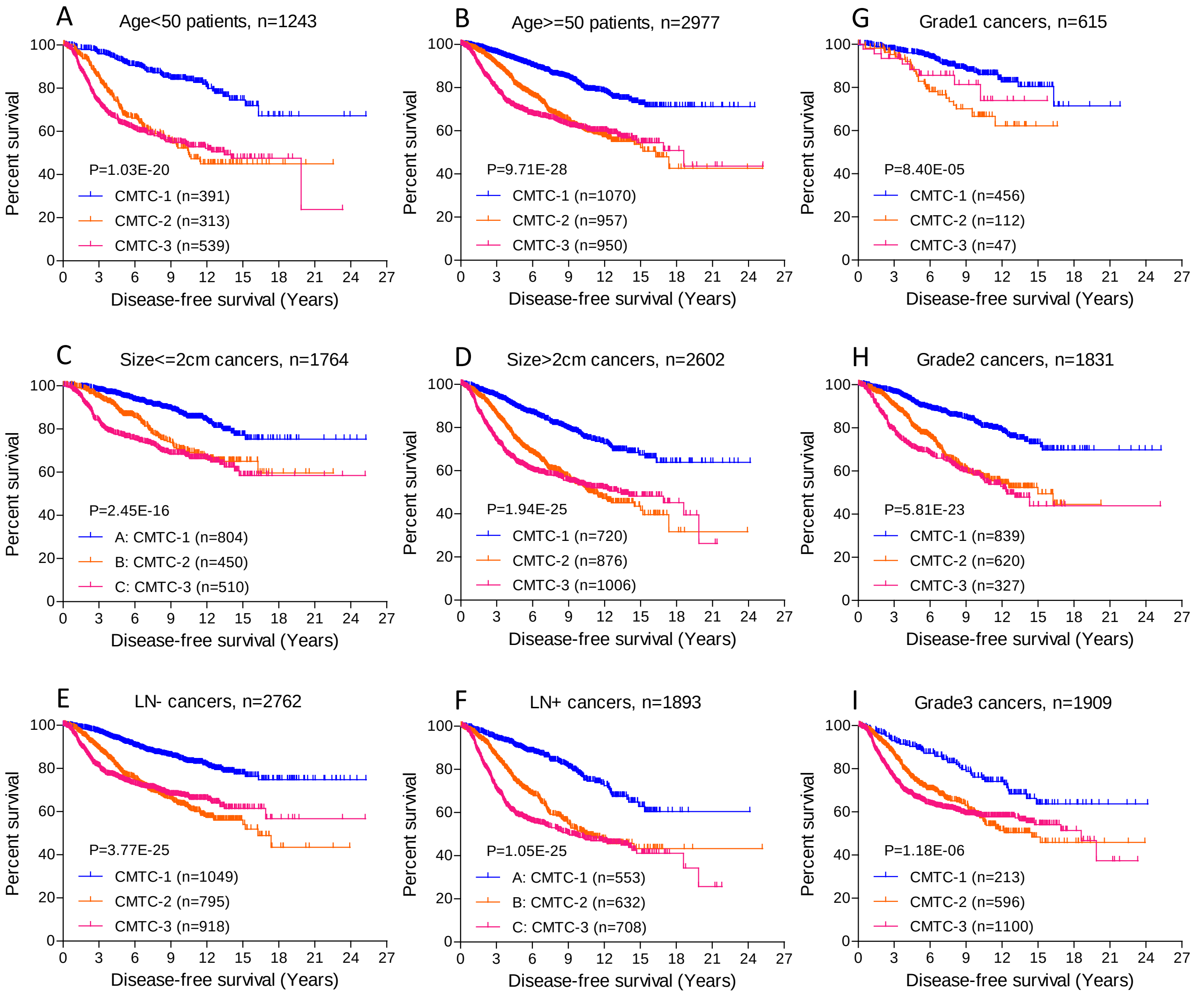

Supplementary Figure S5. The assessment of the prognostic significance of *CMTC* among different groups based on different clinical variable status in the 4851 overall breast cancers. CMTC prognostic predictions between different age of the patient: (**A**) Age < 50 patients and (**B**) Age >= 50 patients; different tumor size: (**C**) tumor size <= 2cm and (**D**) tumor size > 2cm; different lymph node status: (**E**) lymph node negative and (**F**) lymph node positive; and different tumor grade: (**G**) Grade1, (**H**) Grade2 and (**I**) Grade3. The *P* values were determined using Log-rank test based on overall comparison.

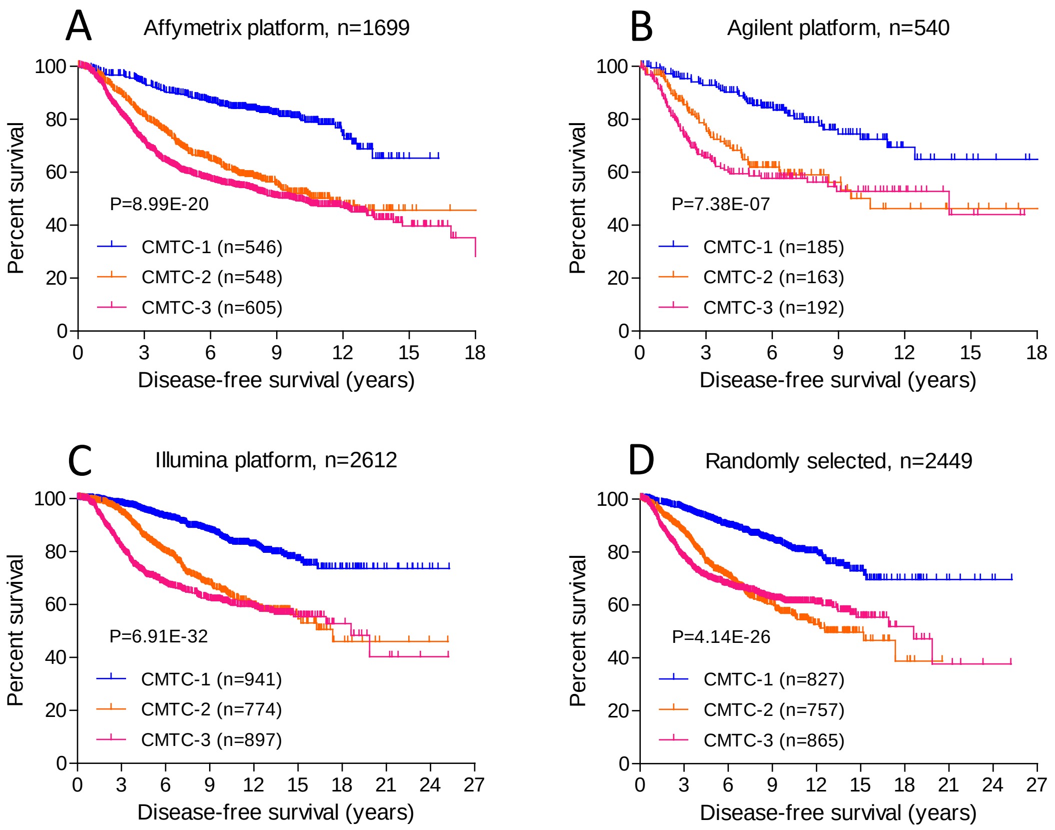

Supplementary Figure S6. The assessment of the prognostic significance of *CMTC* among different groups based on the different microarray platforms in the 4851 overall breast cancers. The overall cohort combined by all internal and external patients. CMTC prognostic predictions in different microarray platforms: (**A**) Affymetrix GeneChip, (**B**) Agilent oligonucleotide microarrays, (**C**) Illumina BeadChip, and (**D**) Randomly selected microarrays. The *P* values were determined using Log-rank test based on overall comparison.
